# Characterization of a novel RNAi yeast insecticide that silences mosquito *5-HT1 receptor* genes

**DOI:** 10.1038/s41598-023-49799-3

**Published:** 2023-12-15

**Authors:** Keshava Mysore, Teresia M. Njoroge, Akilah T. M. Stewart, Nikhella Winter, Majidah Hamid-Adiamoh, Longhua Sun, Rachel Shui Feng, Lester D. James, Azad Mohammed, David W. Severson, Molly Duman-Scheel

**Affiliations:** 1 Department of Medical and Molecular Genetics, Indiana University School of Medicine, Raclin-Carmichael Hall, 1234 Notre Dame Ave., South Bend, IN 46617 USA; 2 The University of Notre Dame Eck Institute for Global Health, Notre Dame, IN USA; 3 Department of Life Sciences, Faculty of Science & Technology, The University of the West Indies, St. Augustine Campus, Trinidad and Tobago, Spain; 4 Department of Biological Sciences, The University of Notre Dame, Notre Dame, IN USA

**Keywords:** Development, RNAi, Infectious diseases, Biotechnology, Developmental biology, Entomology

## Abstract

G protein-coupled receptors (GPCRs), which regulate numerous intracellular signaling cascades that mediate many essential physiological processes, are attractive yet underexploited insecticide targets. RNA interference (RNAi) technology could facilitate the custom design of environmentally safe pesticides that target GPCRs in select target pests yet are not toxic to non-target species. This study investigates the hypothesis that an RNAi yeast insecticide designed to silence mosquito *serotonin receptor 1 (5-HTR1)* genes can kill mosquitoes without harming non-target arthropods. 5-HTR.426, a *Saccharomyces cerevisiae* strain that expresses an shRNA targeting a site specifically conserved in mosquito *5-HTR1* genes, was generated. The yeast can be heat-inactivated and delivered to mosquito larvae as ready-to-use tablets or to adult mosquitoes using attractive targeted sugar baits (ATSBs). The results of laboratory and outdoor semi-field trials demonstrated that consumption of 5-HTR.426 yeast results in highly significant mortality rates in *Aedes, Anopheles,* and *Culex* mosquito larvae and adults. Yeast consumption resulted in significant *5-HTR1* silencing and severe neural defects in the mosquito brain but was not found to be toxic to non-target arthropods. These results indicate that RNAi insecticide technology can facilitate selective targeting of GPCRs in intended pests without impacting GPCR activity in non-targeted organisms. In future studies, scaled production of yeast expressing the 5-HTR.426 RNAi insecticide could facilitate field trials to further evaluate this promising new mosquito control intervention.

## Introduction

Mosquito vector control is the primary strategy for prevention and management of mosquito-borne illnesses^1^, including arboviral diseases and malaria. However, due to the rapid spread of mosquito resistance to commonly used chemical insecticides, the existing set of tools for vector control is insufficient^2^. The potential impacts of chemical insecticides on public health and the environment^3–5^ are also major concerns, and discovery of novel effective and environmentally safe vector control interventions is therefore paramount. The insect central nervous system (CNS) is the primary target for all four classes of insecticides, including pyrethroids^6^, organophosphates, carbamates^7^, and organochlorines^8^. Prolonged opening of sodium channels or neuronal overstimulation leads to death of the insect, and as such the CNS provides an avenue to explore novel insecticide design targets using genetic approaches.

The receptors of key signaling neuroactive molecules (biogenic amines) in the CNS, peripheral nervous system, and non-neuronal tissues of both vertebrates and invertebrates, including serotonin (5-hydroxytryptamine; 5-HT), dopamine (DA), and octopamine (OA)^9^ are excellent pesticide targets. Mosquitoes have orthologs of mammalian biogenic amine receptors, including dopamine, octopamine and serotonin receptors belonging to Class A GPCRs^10^**.** GPCRs, characterized by seven transmembrane domains, are a large group of conserved proteins on the surfaces of animal cells that detect extracellular signals and transmit these signals inside the cell^11,12^. In humans, GPCRs are exploited pharmacologically in a variety of pathological conditions and are the target for ~ 34% of pharmaceutical drugs^13–15^. In insects, these receptors are involved in various biological processes including development, locomotion, reproduction and feeding and are hence important, yet relatively unexplored targets for novel insecticides^16^.

Functional characterization of mosquito-specific GPCR families continues to unravel promising novel insecticide targets that do not impact other organisms**.** Exploring arthropod GPCRs as new putative insecticide targets is a research focus of current interest. The advancement of next generation sequencing, which has yielded high-quality genome assemblies in mosquitoes, continues to facilitate the identification of GPCRs for functional and pharmacological analysis. Among the mostly studied GPCRs in mosquitoes are dopamine receptors, the insecticidal targeting potential of which has been described in *Aedes aegypti* and* Aedes albopictus*^17–19^, *Culex quinquefasciatus*^20^, *Anopheles gambiae*^19,21,22^, and other insects including *Drosophila melanogaster*^23,24^ and *Tribolium castaneum*^25^.

5-HT receptors, the largest group of GPCRs, are located on the cell membrane of neurons and other cell types. The serotonin receptors are known to share significant orthology in a diverse range of organisms and mediate the effects of 5-HT as the endogenous ligand^26^. 5-HT is an important neurotransmitter molecule that is distributed widely in the central and the peripheral nervous systems in animal phyla and is involved in a wide range of physiological processes including sleep, thermoregulation, memory, learning, pain, aging, motor activity, longevity, biological rhythms, and feeding among other functions^27–32^. In both vertebrates and invertebrates, serotonin acts as a ligand on many organ systems through seven families of serotonin receptors (5-HTR1 − 7) that constitute numerous receptor isoforms^33,34^. The insect 5-HT receptors have different pharmacological characteristics compared to their vertebrate counterparts and may utilize varying modes of signal transduction^34^. In insects*,* 5-HT receptors are known to modulate sleep, feeding, the circadian clock, and learning and memory, and thus regulate several insect behaviors among other physiological processes^34^. Although few data about pharmacological properties of insect 5-HT receptors are available, these properties are currently being elucidated in various insects^34^.

The 5-HT1 receptor family, a heterogenous group of serotonin receptors with various subtypes including 5-HTR1A, 5-HTR1B, 5-HTR1D, 5-HTR1E and 5-HTR1F receptors, are involved in inhibition of adenylyl cyclase opening of K^+^ channels in the CNS and are conserved in dipteran insects including mosquitoes^35^. In the fruit fly *D. melanogaster,* putative 5-HTR1A and 5-HTR1B subtypes are found in the larval CNS and are known to regulate aggression, sleep, olfactory learning, memory, feeding, circadian entrainment behavior and larval locomotion^36^. Blocking the activity of 5-HTR1B and 5-HTR2B was reported to interfere with the immune system and enhance insecticide sensitivity in flies^37^. In mosquitoes, 5-HTRs are present in all stages of mosquito development, with elevated expression levels in the adult stage^10^ playing a vital role in regulation of the immune and nervous systems among other physiological functions. Pharmacological studies to agonize or antagonize 5-HTRs have demonstrated their physiological functions in mosquitoes. In *A. aegypti* and *A. gambiae*, the dysfunction of 5-HTRs was reported to adversely affect adult locomotion (inhibition of flight performance) and blood-feeding behavior, identifying the 5-HTRs as potential insecticide targets for the disease vectors^10^. CRISPR-Cas9-mediated silencing of *5-HTR2B* in adult *A. aegypti* was recently shown to inhibit growth through reduced lipid accumulation^38^, whereas the deletion of *5-HTR7A* resulted in significant larval and pupal mortality^39^. More recently, inhibition of the serotonergic system in adult *A. aegypti* altered male phonotaxis, a behavior that’s associated with hearing impairment and has implications on mating behavior^40^. The results from these few functional studies of mosquito 5-HTRs are beginning to unravel their potential as novel putative insecticide targets, highlighting the need for further functional analyses of *5-HTR* genes in mosquitoes.

Based on previous functional characterizations of 5-HTRs in *A. aegypti* and *A. gambiae*^10,38–40^, it was hypothesized that silencing of *5-HTR1* in multiple disease vector mosquito species would result in significant larval and adult mortality. Recently, we have characterized and developed several species-specific interfering RNA pesticides for larval and adult mosquito control through RNAi-mediated gene silencing using modified baker’s yeast as the delivery system^19,41–43^. In this study, we developed and characterized a yeast RNAi pesticide targeting *5-HTR1* genes in larvae and adult *A. aegypti*, *A*. *albopictus*, *A. gambiae*, *Culex pipiens* and *C. quinquefasciatus*. In all the species, the larvae were fed with yeast expressing shRNAs matching the *5-HTR1* gene, whereas for adults, the same RNAi yeast was mixed with an attractive sugar bait (ASB) and delivered orally. Silencing of *5-HTR1* via RNAi resulted in significant larval and adult mosquito mortality, revealing *5-HTR1* genes as excellent novel putative insecticide targets for biorational control of disease vector mosquitoes.

## Results and discussion

### Silencing 5-HTR leads to mortality in *Culicine* and *Anopheline* larvae and adults

A microinjection screen for insecticides that target *A. aegypti* genes required for survival at multiple stages of the mosquito life cycle identified siRNA 5-HTR.426. The nucleotide target sequence of 5-HTR.426 siRNA, which is located in the *A. aegypti* gene *AAEL000528*, is conserved across various *Aedes*, *Anopheles,* and *Culex* mosquito species, but an identical target nucleotide sequence has not been found outside of mosquitoes to date (Supplementary Table S1). The conserved nucleotide sequence might serve as a target site for a mosquito-specific insecticide that could be utilized as a valuable tool for biorational mosquito control while not causing any harm to non-target organisms. When adult female *A. aegypti* and *A. gambiae* were injected with 5-HTR siRNA, it resulted in significant mortality rates of 87 ± 8% (P = 1.67844E−19, compared to control siRNA treatment) and 48 ± 4% (P = 4.0455E−10, compared to control siRNA treatment, Table 1), respectively. Furthermore, immersing L1 larvae of *A. aegypti* and *A. gambiae* in 5-HTR.426 siRNA led to significant larval mortality, with rates of 35 ± 0% (P < 0.001) and 63 ± 2% (P < 0.001), respectively, compared to control siRNA treatment, (Table 1). These findings support the hypothesis that 5-HTR.426 siRNA exhibits both adulticidal and larvicidal activity in both culicine and anopheline mosquitoes.

**Table 1:** Mortality induced by 5-HTR.426 siRNA.

Experiment	Mortality ± SD (%)				
	Species	Control siRNA	5-HTR siRNA	P-value	n
Larval soaking	*A. aegypti*	0 ± 0	35 ± 0	1. 5E^−05^	40
	*A. gambiae*	5 ± 0	63 ± 2	7.0E^−11^	40
Adult injection	*A. aegypti*	8 ± 2	87 ± 8	1.7E^−19^	60
	*A. gambiae*	2 ± 2	48 ± 4	4.0E^−10^	60

The average percent mortality rates for *A. aegypti* and *A. gambiae* larvae and adults are shown along with the standard deviations (SDs) for the soaking treatments and the standard errors of the mean (SEMs) for the injection treatments. The total numbers of individuals subjected to each treatment (n) and p values obtained from Fisher’s exact test analyses between 5-HTR.426 siRNA-treated and corresponding control siRNA-treated individuals are also shown.

*AAEL000528* encodes a *5-HTR1* ortholog. Phylogenetic analyses performed at the amino acid level indicate that the amino acid sequence of the protein encoded by *AAEL000528* has > 70% sequence identity among the 5-HTR1 mosquito orthologs and < 40% sequence identity to the distantly related Drosophilids (Supplementary Fig. S1). The mosquito 5-HTR1 ortholog protein sequences are more similar to each other than to any of the *Drosophila* 5-HTR1 proteins (Supplementary Fig. S2).

### High levels of mortality are induced in larvae by consumption of 5-HTR.426 yeast

Next, we capitalized on the many assets of *S. cerevisiae* (baker’s yeast), which has been developed as an expression and delivery system for interfering RNA that silences genes in a sequence-specific manner^44,45^. Yeast, which is often a component of larval rearing diets^46,47^, is generally safe and highly attractive to both larvae and adult mosquitoes^48^, allowing for the development of safe and effective mosquito insecticides. Based on the screening results, a non-integrating multi-copy yeast shuttle plasmid containing a shRNA corresponding to the 5-HTR.426 siRNA target sequence, the expression of which was placed under control of a constitutive promoter, was created. Baker’s yeast *S. cerevisiae*, transformed with this plasmid, hereafter referred to as 5-HTR.426 yeast, was cultured and used to prepare heat-inactivated yeast, which was hypothesized to have insecticidal properties. Heat-inactivated lyophilized 5-HTR.426 yeast was fed to L1 larvae of various *Aedes, Anopheles,* and *Culex* mosquito species (Table 2), which continued to consume it throughout larval development.

**Table 2:** Mortality induced by 5-HTR.426 yeast.

Species	Mortality ± SEM (%)			
	Control	5-HTR.426	P-value	n
*A. aegypti*	1 ± 1	87 ± 1	3.1^–17^	180
*A. albopictus*	1 ± 1	90 ± 1	4.0E^−17^	180
*A. gambiae*	1 ± 1	91 ± 1	5.6E^−16^	180
*C. quinquefasciatus*	2 ± 1	89 ± 1	1.1E^−14^	180
*C. pipiens*	13 ± 3	90 ± 1	4.0E^−09^	180

The average percent mortality rates for *A. aegypti*, *A. albopictus*, *A. gambiae*, *C. quinquefasciatus* and *C. pipiens* larvae that were fed with 5-HTR.426 insecticidal or control yeast are shown. The standard errors of the mean (SEMs) along with the total numbers of individuals subjected to each treatment (n) are also provided. The p values obtained from Student’s t-test for the arcsine transformed data between 5-HTR.426-treated and corresponding control-treated individuals are also shown.

**Figure 1:**
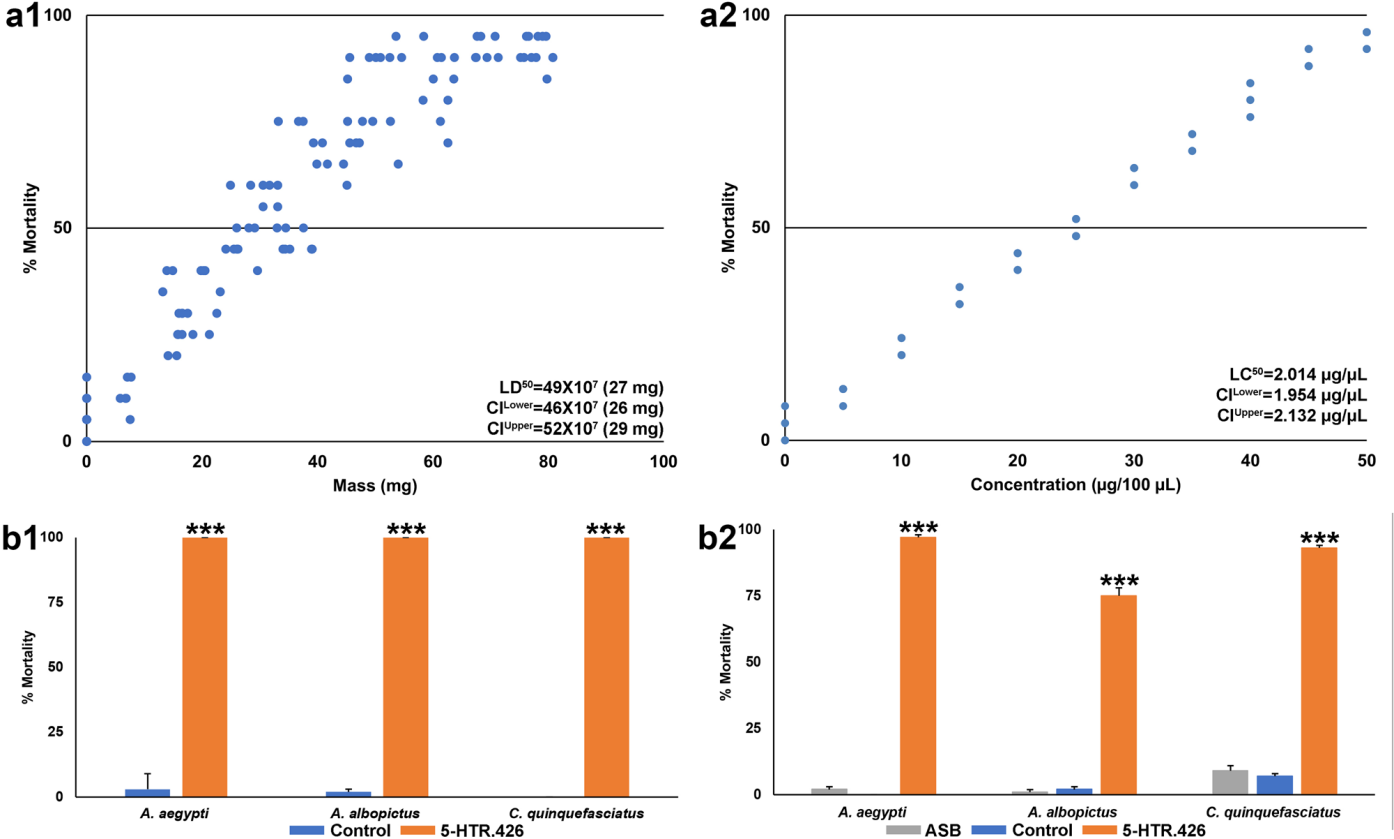
5-HTR.426 shRNA-expressing yeast induces mortality under laboratory and semi-field conditions. (**a1**) In laboratory experiments, yeast expressing 5-HTR.426 shRNA induced dose-dependent mortality in the *A. aegypti* LVP-IB12 laboratory strain. The LD_50_ (for treatment of 20 larvae) was 27 mg. (**b1**) When the same yeast was applied to mosquito strains collected from the field and tested in semi-field conditions in Trinidad, it eliminated all the larvae in the experiment (orange bars). (**a2**) In laboratory trials, 5-HTR.426 yeast induced dose-dependent mortality in *A. aegypti* LVP-IB12 adult females (LC_50_ = 2.014 ug/ul). (**b2**) In outdoor semi-field trials conducted in Trinidad, 5-HTR.426 yeast also induced significant mortality in field strains of *Aedes* and *Culex* mosquitoes. The data in (**b1**) and (**b2**) are presented as mean percentage mortality, with error bars representing standard error of the mean. *** indicates a statistically significant difference from the control group (P < 0.001). ASB, attractive sugar bait alone; control refers to treatments with control RNAi yeast.

Although larvae ingesting control yeast^48^ survived, ~ 90% of those consuming the interfering RNA 5-HTR.426 yeast died in indoor laboratory trials (Table 2), indicating that the yeast is significantly (P < 0.001) larvicidal to *A. aegypti, A. albopictus, A. gambiae, C. pipiens,* and *C. quinquefasciatus* mosquitoes. Although deletion of the 5-HT7A receptor resulted in larvae with aberrant head-to-chest ratios and decreased motility^39^, neither phenotype was observed in 5HTR.426-treated larvae. Likewise, although disruption of the *A. aegypti* 5HT2B receptor resulted in reduced larval size^38^, this phenotype was not observed following the silencing of *5-HTR1* in any of the mosquito larval species. These data suggest that this receptor does not impact insulin signaling as reported in other 5-HTR subtypes^49–52^. When 5-HTR.426 yeast was supplied beginning in the late third instar, no significant larval death was observed (P > 0.05), suggesting that treatment beginning in early larval development is critical for 5-HTR.426 larvicidal activity. When treatments initiated in L1, the majority of 5-HTR.426-treated larvae died within eight days by the fourth larval instar, whereas the control yeast-fed larvae survived until adulthood. The percentage of larval mortality observed correlated with the dosage of 5-HTR.426 (Fig. 1a), with the LD_50_ for *A. aegypti* larvae determined to be 27 ± 2 mg per 20 larvae.

The impact of 5-HTR.426 yeast on survival of mosquito larvae suggested that it may function as an effective larvicide for targeting multiple species of mosquitoes that spread arboviral and parasitic diseases across the globe. To this end, it is important to demonstrate that the yeast can be shipped globally, ideally at room temperature storage conditions, without losing activity, and that the shipped yeast retains its insecticidal activity. Moreover, it is important to evaluate the effectiveness of the yeast in field-collected mosquito strains under natural tropical conditions in disease-endemic countries.

To further evaluate the effectiveness of the yeast in tropical settings following international shipment, yeast produced in Indiana was shipped at room temperature to a trial site in Trinidad, where semi-field trials were conducted at the University of the West Indies, St. Augustine, Trinidad and Tobago. These experiments, which included evaluation of locally collected *A. aegypti, A. albopictus,* and *C. quinquefasciatus* mosquitoes, resulted in 100% mortality of newly hatched larvae (Fig. 1b1). Moreover, the larvae died within one day of treatment, indicating that field strains treated in natural tropical habitats may be more susceptible to the insecticides than those reared under laboratory conditions (26.5 °C), which do not reach the same high temperatures attained in an outdoor rooftop laboratory in Trinidad (up to 32.0 °C in these trials). These studies suggest that yeast RNAi pesticides have the potential for successful field implementation. However, this will need to be confirmed in larger-scale field trials, which should focus on the residual activity of RNAi yeast following field deployment. This is a critical factor that must be evaluated in order to fully assess the potential of these insecticides as a new mosquito control intervention.

### Application of ATSBs to deliver 5-HTR.426 in field simulated conditions

Attractive targeted sugar bait (ATSBs) technology is emerging as a promising new mosquito control method that involves luring mosquitoes to feed on a sugar lure containing a poison. ATSBs have the potential to reduce mosquito densities, as well as arboviral disease and clinical malaria incidences, when used in conjunction with existing vector control strategies^53^. The impact of ATSBs on malaria incidence is presently being assessed in a Phase 3 clinical trial which is being conducted in three African nations^54^. Although the insecticides assessed in ATSBs to date have included a variety of broad-based insecticides, RNAi insecticides^55^, which can be custom-designed to target species of interest, could significantly advance ATSB technology^53^. The adulticidal activity of 5-HTR.426 yeast was therefore assessed. This was initially pursued using a previously described lab-based sucrose feeder system^43^, and then in conjunction with a miniature bait station sachet system designed to mimic the commercial bait stations that are presently being evaluated in the phase 3 clinical trials^54^.

Both delivery systems were assessed in *A. aegypti, A. albopictus, C. quinquefasicauts, C. pipiens* and *A. gambiae*. In all cases, significant morbidity (P < 0.001, Tables 3, 4) was observed with respect to both sugar bait alone or with control RNAi yeast^48^. An average of 85% mortality was observed in conjunction with the lab-based sucrose feeder system (Table 3), while over 95% morbidity was observed when utilizing the commercial bait system (Table 4). 85% or higher feeding rates were observed in all yeast treated groups (Table 5), and 100% feeding rates were observed in all commercial bait station system trials (Table 5). No significant differences were observed between the control yeast and 5-HTR.426 treatment groups (P > 0.05), though a significantly lower feeding rate (P < 0.05) was observed in *C. pipiens* field strain mosquitoes treated with sucrose bait alone (Table 5), suggesting that the addition of yeast to sucrose bait may increase feeding rates in field strains, a phenomenon that will be assessed in future field studies.

**Table 3:** Lab trials examining the mortality induced by 5-HTR.426 yeast-ATSB in various mosquitoes.

Species	Mortality ± SEM (%)				
	ASB	Control	5-HTR.426	P-value	n
*A. aegypti*	1 ± 1	2 ± 1	88 ± 1	< 0.00001	180
*A. albopictus*	0 ± 0	0 ± 0	85 ± 2	< 0.00001	225
*A. gambiae*	1 ± 1	1 ± 1	94 ± 1	< 0.00001	180
*C. quinquefasciatus*	1 ± 1	1 ± 1	91 ± 1	< 0.00001	225
*C. pipiens*	2 ± 1	2 ± 1	82 ± 3	< 0.00001	225

The average percent morbidity rates for *A. aegypti*, *A. albopictus*, *A. gambiae*, *C. quinquefasciatus* and *C. pipiens* adult females that were fed with lab attractive sugar bait (ASB) alone and yeast expressing the 5-HTR.426 or control shRNA are shown. The trials were performed in the insectary. The standard errors of the mean (SEMs) along with the total numbers of individuals subjected to each treatment (n) are provided. The p values obtained from one-way ANOVA conducted between individuals fed with attractive sugar bait alone (ASB) and 5-HTR.426- or control-yeast treated insects are provided.

**Table 4:** Delivery of RNAi yeast ATSB in a miniature sachet commercial bait system simulated field trial.

Species	Mortality ± SEM (%)				
	ASB	Control	5-HTR.426	P-value	n
*A. aegypti*	8 ± 1	6 ± 1	97 ± 2	< 0.00001	225
*A. albopictus*	6 ± 1	6 ± 2	96 ± 1	< 0.00001	225
*A. gambiae*	6 ± 2	12 ± 4	98 ± 1	< 0.00001	180
*C. quinquefasciatus*	4 ± 2	4 ± 2	100 ± 0	< 0.00001	225
*C. pipiens*	2 ± 1	3 ± 1	99 ± 1	< 0.00001	225

The average percent morbidity rates for *A. aegypti*, *A. albopictus, A. gambiae*, *C. quinquefasciatus* and *C. pipiens* adult females that were fed with commercial attractive sugar bait (ASB) alone, bait containing yeast expressing 5-HTR.426 (5-HTR.426) or bait containing control yeast (control) within the miniature sachet membrane feeding systems are shown. The standard errors of the mean (SEMs) along with the total numbers of individuals subjected to each treatment (n) are also provided. The p values obtained from one-way ANOVA between individuals fed with ASB alone, 5-HTR.426 or control-treated adult mosquitoes are provided.

**Table 5:** RNAi yeast ATSB feeding rates.

Experiment	Species	Feeding rate ± SEM (%)				
		ASB	Control	5-HTR.426	P-value	n
Lab Sugar Bait	*A. aegypti*	100 ± 0	100 ± 0	100 ± 0	1	180
	*A. albopictus*	87 ± 3	89 ± 5	85 ± 4	0.2490	225
	*A. gambiae*	100 ± 0	100 ± 0	100 ± 0	1	180
	*C. quinquefasciatus*	100 ± 0	100 ± 0	100 ± 0	1	225
	*C. pipiens*	68 ± 3	88 ± 1	89 ± 3	3.8E^−15^	225
Westham Co.Bait	*A. aegypti*	100 ± 0	100 ± 0	100 ± 0	1	225
	*A. albopictus*	100 ± 0	100 ± 0	100 ± 0	1	225
	*A. gambiae*	100 ± 0	100 ± 0	100 ± 0	1	180
	*C. quinquefasciatus*	100 ± 0	100 ± 0	100 ± 0	1	225
	*C. pipiens*	100 ± 0	100 ± 0	100 ± 0	1	225

The percentages of insects that became engorged with sugar meals consisting of each of the indicated treatments are shown in the table. The feeding system, mosquito species, feeding rates with standard errors of the mean (SEMs), and the total number of individuals subjected to each treatment (n) are also shown. The p-values obtained by carrying out a one-way ANOVA on all treatments are provided. No significant differences were observed between the attractive sugar bait alone (ASB), control (bait with control yeast) and bait with 5-HTR.426 yeast insecticide (5-HTR.426) treatments.

**Figure 2:**
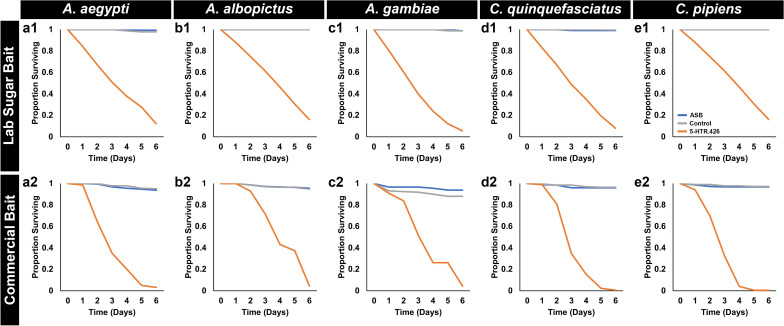
Yeast expressing 5-HTR.426 shRNA results in high levels of adult mortality. Female mosquitoes of five different species were fed with 5-HTR.426 shRNA expressing yeast either using the lab sugar bait system (laboratory trials shown in (**a1**–**e1**)) or commercial bait system (simulated field trials shown in (**a2**–**e2**)). The yeast induced high levels of mortality over 6 days post feeding. Individuals fed with yeast mixed with commercial bait die 24–36 h earlier than those fed with lab sugar bait with yeast. ASB, attractive sugar bait alone; control refers to treatments with control RNAi yeast.

Mosquitoes fed with either Westham ASB alone or with control yeast showed negligible mortality in both cases (Tables 3, 4). Among the individuals treated with 5-HTR.426, mortality occurred predominantly within 4–5 days when using lab-based bait (Fig. 2a1–e1), while those fed through the commercial bait system experienced slightly earlier mortality by days 3–4 (Fig. 2a2–e2). Differences in the size of the sugar meal could explain these results, which suggest that the mosquitoes are equally attracted to both sucrose and commercial baits, but that they may drink more of the Westham bait, resulting in shorter dying times. Finally, the percentage of morbidity correlated with the concentration of 5-HTR.426 shRNA in the yeast, as evidenced by feeding *A. gambiae* mosquitoes with various concentrations of yeast in the laboratory sucrose and commercial bait systems, for which the LC_50_ values were determined to be 2.014 μg yeast/μL sucrose solution (Fig. 1a2) and 1.9924 μg yeast/μL ATSB, respectively.

The 5-HTR.426 yeast ATSB sachets were also evaluated in outdoor semi-field trials conducted in Trinidad. 5-HTR.426 yeast ATSB induced significant morbidity (P < 0.001) in field strains of *A. aegypti, A. albopictus, and C. quinquefasciatus* mosquitoes in outdoor rooftop semi-field trials conducted in Trinidad (Fig. 1b2). As observed in the larvicide trials (Fig. 1b1), this morbidity typically occurred within the first day of treatment, faster than death rates observed in the laboratory, which occurred within 2–6 days following treatment (Fig. 2).

### 5-HTR.426 yeast evaluation in non-target arthropods

Application of 5-HTR.426 yeast was demonstrated to kill mosquitoes but given that the target site for this yeast is not conserved outside of mosquitoes (Supplementary Table S1), it is predicted to have little if any impact on non-target organisms. To assess this, the yeast was fed to a variety of non-target arthropods (Table 6). No significant mortality was observed following consumption of control vs. 5-HTR.426 yeast by select non-target arthropods, including *D. melanogaster, T. castaneum, Oncopeltus fasciatus,* and *Hippodamia convergens* adults. These results suggest that the yeast insecticide is specific to mosquitoes. Although unlikely to occur, potential impacts on non-target organisms could be further reduced through deployment of the yeast in conjunction with the commercial membrane evaluated in the commercial bait system trials, which have incorporated a perforated membrane through which only mosquitoes can probe to access the sugar meal and the active ingredient, further reducing risks to non-target organisms, including pollinators^56^. Furthermore, this new generation of RNAi ATSBs could be used in combination with other interventions as part of integrated vector management and to circumvent insecticide resistance to various traditional synthetic pesticides.

**Table 6:** 5-HTR.426 yeast has no significant effect on non-target arthropod survival.

Test Organism	% Survival ± SD			
	Developmental stage	n/Treatment	Control yeast	5-HTR.426
*D. melanogaster*	Larvae	40	93 ± 4	98 ± 4
*D. melanogaster*	Adult	40	95 ± 7	98 ± 4
*T. castaneum*	Adult	80	96 ± 5	98 ± 3
*O. fasciatus*	Adult	60	85 ± 9	88 ± 6
*H. convergens*	Adult	40	90 ± 8	95 ± 6

The mean percentages of survival following treatments with control or 5-HTR.426 insecticidal yeast (5-HTR.426). The number of animals treated (n) in these laboratory trials is also indicated. Fisher's exact test comparisons did not reveal any significant differences in survival between 5-HTR.426 insecticidal yeast-treated (5-HTR.426) and control interfering RNA yeast (control yeast)-treated arthropods (P > 0.05).

### Silencing 5-HTR1 leads to neural defects in the serotonergic pathway in *A. aegypti*

**Figure 3:**
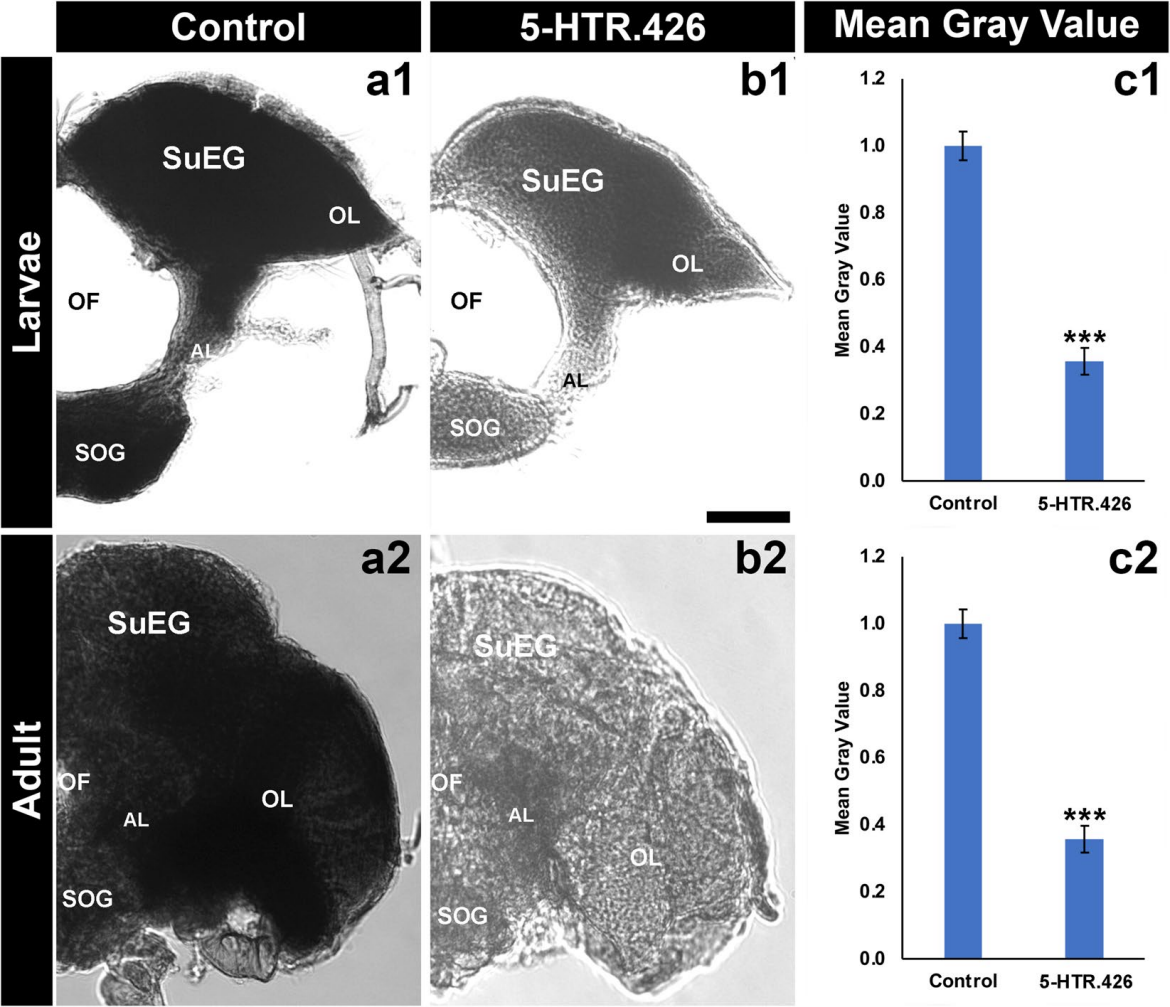
5-HTR.426 yeast results in silencing of the target transcript. Consumption of yeast expressing 5-HTR.426 throughout larval life results in significant reduction of *5-HTR1* transcripts in *A. aegypti* larvae (**a1**–**c1**). *A. aegypti* adults fed with ASB (lab bait) containing 5-HTR.426 yeast induces a similar reduction in the transcript levels when compared with individuals fed with the control yeast (control; **a2**–**c2**). *** = P < 0.001 vs. control yeast; data were analyzed with Student’s t-test. Scale Bar = 100 μm.

Given the potential application of 5-HTR.426 yeast as insecticides, the mode of action was characterized in further detail, a requirement for any future registry applications. Insect GPCRs have indispensable functions in insect physiology. Based on the known CNS defects observed in insects lacking proper function of 5-HTRs^36,57^, it was hypothesized that silencing *5-HTR1* expression would impact mosquito neural functions. *5-HTR1* expression was detected throughout the *A. aegypti* brain, with higher concentrations in the optic lobe and suboesophageal ganglion of larvae, and the mid-brain and optic lobe in adults (Fig. 3). When larvae consumed 5-HTR.426 yeast, there was a significant reduction of 67 ± 3% in *5-HTR1* transcripts (Fig. 3a1–c1; P < 0.001) in the brain, while adult females fed with 5-HTR.426 ATSB displayed 63 ± 2% reduction in mRNA levels (Fig. 3a2–c2; P < 0.001). These findings indicate that 5-HTR.426 yeast consumption results in significant decreases in 5-HTR1 gene expression in the mosquito brain.

**Figure 4:**
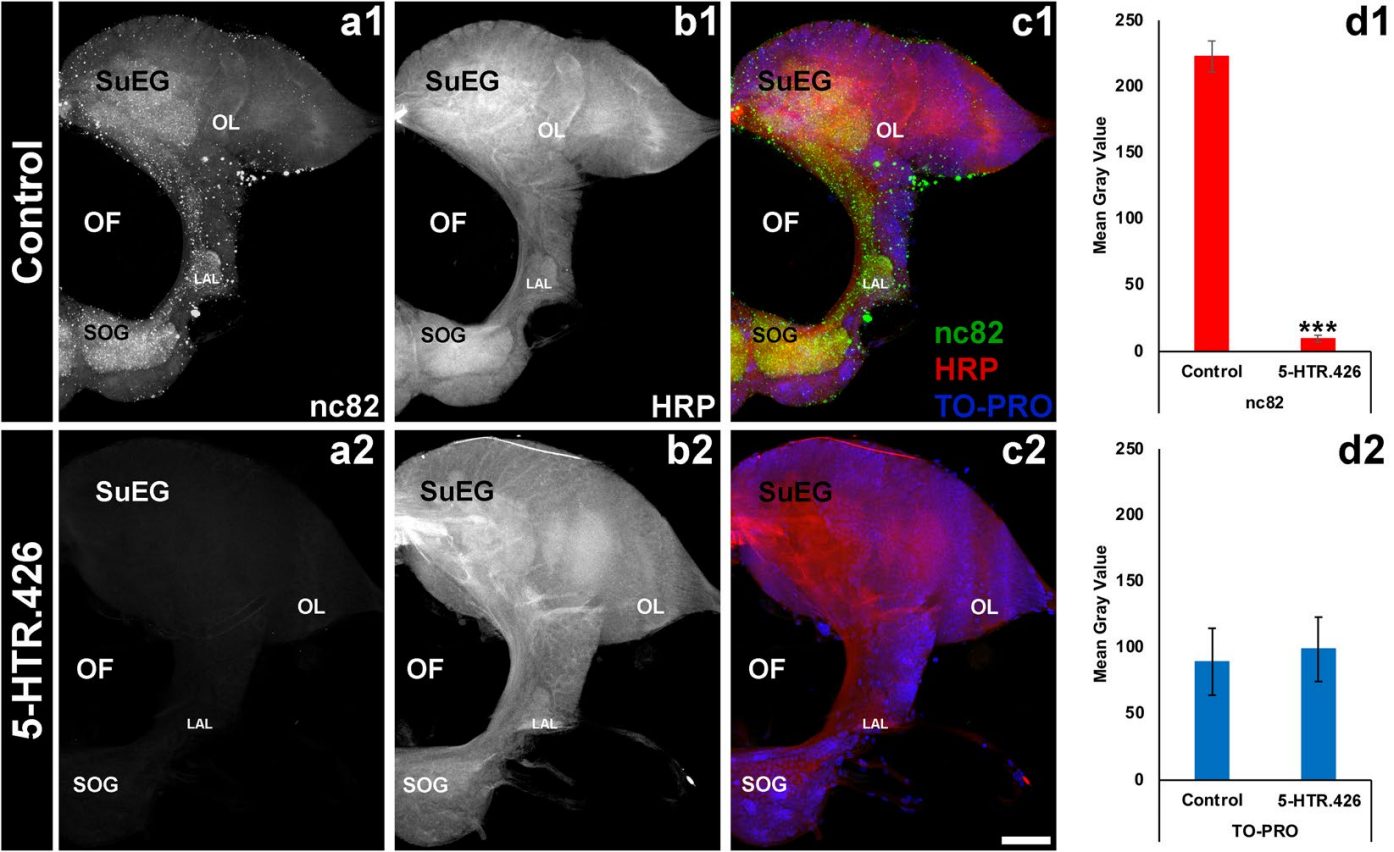
Feeding *A. aegypti* larvae with 5-HTR.426 yeast causes neural defects. Larval brains were labeled with three antibodies: mAbnc82 (an active synapse marker; white in (**a1**, **a2**); green in (**c1**, **c2**)), anti-HRP (neural marker; white in (**b1**, **b2**); red in (**c1**, **c2**)), and TO-PRO (nuclear marker; blue in (**c1**, **c2**)). The levels of nc82, a protein involved in neural development, were significantly reduced in the synaptic neuropil of larvae fed with 5-HTR.426 yeast (**a2**, **c2**) compared to control yeast-treated (control) larvae (**a1**, **c1**). The data are presented as average mean gray values, with error bars denoting standard error of the mean. ***Indicates a statistically significant difference from the control group (P < 0.001). Representative larval brains are oriented dorsal upward in this figure. LAL, larval antennal lobe; OL, optic lobe; SOG, sub-esophageal ganglion; SuEG, supraesophageal ganglion. Scale Bar = 100 μm.

**Figure 5:**
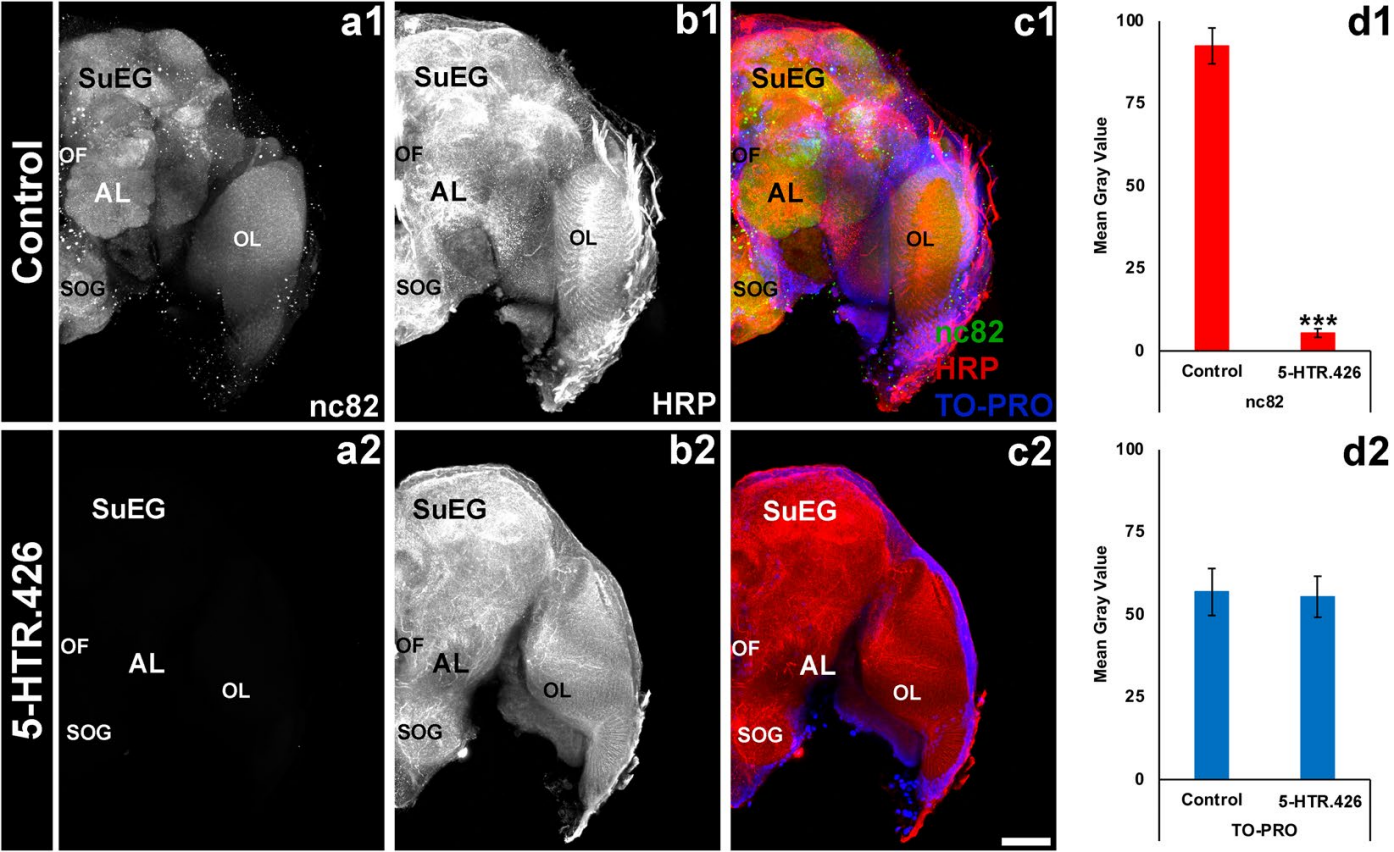
5-HTR.426 yeast induces neural defects in *A. aegypti* adult females. Adult brains of female *A. aegypti* mosquitoes that were fed with either control yeast (**a1**–**c1**) or 5-HTR.426 yeast (**a2**–**c2**), were immunolabeled with mAbnc82 (an active synapse marker; white in (**a1**, **a2**); green in (**c1**, **c2**)), anti-HRP antibodies (a neural marker; white in (**b1**, **b2**); red in (**c1**, **c2**)), and TO-PRO dye (nuclear stain; blue in (**c1**, **c2**)). The levels of nc82 were significantly reduced in the synaptic neuropil of adults fed with 5-HTR.426 (**a2**, **c2** vs. **a1**, **c1**; **d1**) yeast compared to control yeast-treated (control) adults (P < 0.001) while the levels of TO-PRO remained the same in both cases (**d2**). Representative adult brains are oriented dorsal upward in this figure. AL, larval antennal lobe; OL, optic lobe; SOG, sub-esophageal ganglion; SuEG, supraesophageal ganglion. Scale Bar = 100 μm.

To assess if neural activity is affected in 5-HTR.426-treated larvae and adults, we performed nc82 antibody staining, which detects the expression of Bruchpilot, a marker of active neural synapses^58^, following 5-HTR.426 yeast feeding in *A. aegypti* larvae and adults. Although neural density in the brain (Figs. 4d2, 5d2; quantified by TO-PRO nuclear staining) was not significantly different between 5-HTR.426 and control-treated larval or adult brains (Figs. 4d1, 5d1; P > 0.05, t-test: two-tailed, equal variance), nc82 levels were significantly reduced by 95 ± 2% in larval (Fig. 4a1, c1 vs. Fig. 4a2, c2; P < 0.001 vs. control-treated) and 93 ± 3% in adult (Fig. 5a1, c1 vs. Fig. 5a2, c2; P < 0.001 vs. control-treated) brains. These experiments suggest that the mode of action for 5-HTR.426 in *A. aegypti* may involve disruption of neural function, as evidenced by the decrease in Bruchpilot levels in the brains of individuals treated with 5-HTR.426 yeast.

**Figure 6:**
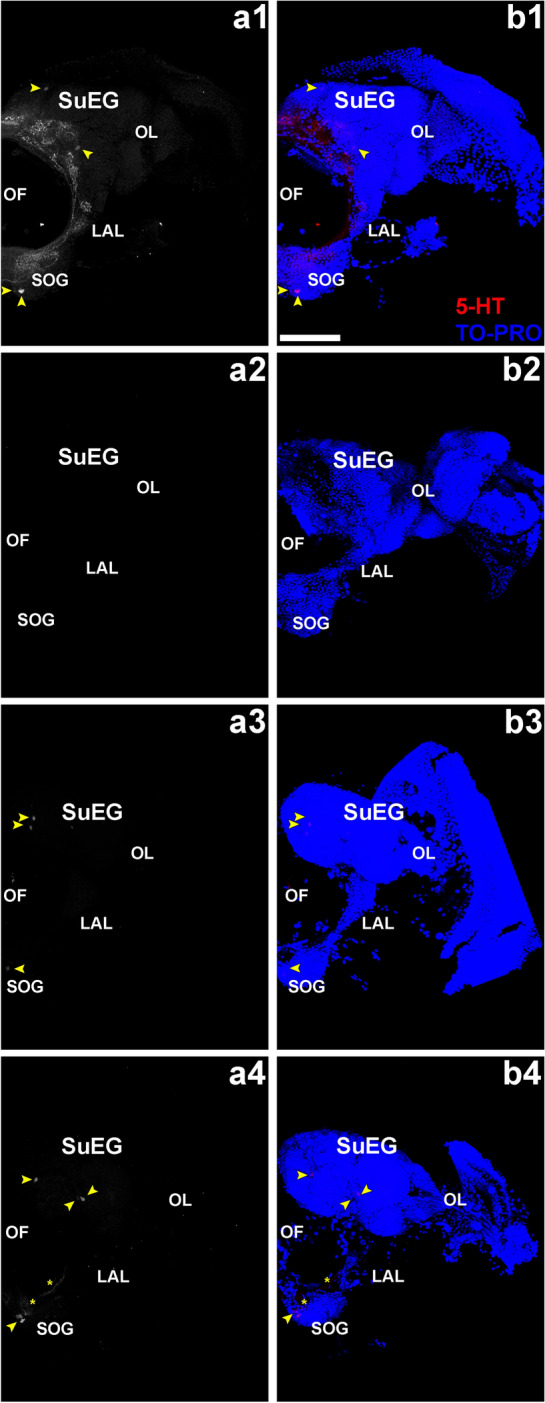
5-HTR.426 yeast induces serotonergic neural defects in the larval brain of 5-HTR.426-treated mosquitoes. In control yeast-fed (control; white in (**a1**); red in (**b1**)) L4 larvae, serotonergic neurons marked by the anti-5-HT antibody (arrowheads) show normal patterns of innervation throughout the larval brain. Individuals treated with 5-HTR.426 insecticidal yeast (**a2**–**a5**) displayed the phenotypes represented here and quantified in Table 7. In ~ 50% of individuals, there is a complete absence of 5-HT neurons (**a2**, **b2**); 36% of treated individuals show 5-HT expression in 2–3 cell bodies (arrowheads) without any dendrite expression (**a3**, **b3**), and the remaining 12% of labeled individuals show expression in 3–4 cell bodies (arrowheads) with variable expression in dendrites (**a4**, **b4**). Representative adult brains are oriented dorsal upward in this figure. AL, larval antennal lobe; OL, optic lobe; SOG, sub-esophageal ganglion; SuEG, supraesophageal ganglion. Yellow arrow heads indicate cell bodies, and yellow asterisks indicate remnant dendrites. Scale bar = 25 mm.

Nerve bundles labeled with anti-HRP showed a small but not statistically significant decrease in intensity (Figs. 4b1, b2; 5b1, b2; P > 0.05 between control and 5-HTR.426) in the area where the sensory neurons synapse with projection neurons, suggesting that specific neural synapses may be disrupted. We therefore assessed if 5-HTR.426 treatments disrupt sensory neural synapses with serotonergic projection neurons. Although larvae fed with control yeast displayed normal 5HT expression, as previously reported^59^ (Fig. 6a1, b1), neural defects were observed in larvae treated with 5-HTR.426 yeast. Approximately 52% of these larvae exhibited a complete loss of 5HT expression in the brain (Fig. 6a2, b2, Table 7), while 36% had expression in only 2–3 cell bodies with no dendritic expression (Fig. 6a3, b3; cell bodies highlighted by yellow arrow heads, Table 7). 12% had expression in 3–4 cell bodies (yellow arrow heads; Fig. 6a4, b4) with some expression in dendrites (yellow asterisk; Fig. 6a4, b4) in the suboesophageal ganglion (SoG; Fig. 6a4), and the remainder showed expression in multiple cell bodies but no dendritic expression (Table 7). These neural defects correlated well with the timing of larval death, suggesting that neural deficits contributed significantly to mosquito mortality.

**Table 7:** Larval brain phenotypes.

Phenotype	% of population
Complete loss of 5-HT expression	52
2–3 cell bodies without dendrites	36
3–4 cell bodies with dendrites	12

The percentage of brains displaying the indicated phenotypes following treatment with 5-HTR.426 yeast is shown. The phenotypes were assessed in early fourth instar larvae labeled with anti-5-HT antibody.

### Conclusion and future prospects

The GPCR family is composed of membrane bound receptors that regulate numerous intracellular signaling cascades which mediate a variety of essential biological processes. As such, GPCRs are attractive yet underexploited pesticide targets, and the identification of environmentally safe pesticides with limited impacts on GPCR function in non-target species is an active area of research^22,60^. Recent advances in RNAi insecticide technology in mosquitoes, coupled with identification of GPCRs in the mosquito genome projects, are facilitating the identification of biorational pesticides that target mosquito GPCRs, yet have little if any impacts on non-target species. We had previously characterized dop1.462, a dual-action larvicidal and adulticidal RNAi pesticide that targets the dopaminergic GPCR encoding-gene *dop1* at a target site that is conserved in multiple species of mosquitoes^19^. Similarly, in this study, we demonstrate that targeting a second GPCR gene, *5-HTR1,* with 5-HTR.426 yeast insecticide results in mosquito larval and adult death (Figs.1, 2). As with the dop1.462 yeast insecticide, the 5-HTR.426 insecticide targets a site conserved in multiple species of mosquitoes that is not found in non-target organisms (Supplementary Table S1). The results of laboratory studies (Figs. 1a, 2a1–e1, Table 1, Table 3), simulated field ATSB trials (performed in the laboratory using the commercial bait and membrane miniature sachet system; Fig. 2a2–e2, Table 4), and semi-field trials (performed with the sachet system in an outdoor enclosure; Fig. 1B) demonstrated that 5-HTR.426 is a highly effective larvicide and adulticide in *Aedes, Anopheles,* and *Culex* mosquitoes. Immunohistochemical analyses revealed significant neural deficits in the larval and adult CNS brain following silencing of 5-HTR.426 (Figs. 3, 4, 5, 6). Despite the severe impacts of 5-HTR.426 consumption on the mosquito nervous system (Figs. 4, 5), the lack of observed toxicity following consumption of the yeast by non-target organisms (Table 5) indicates that this RNAi yeast insecticide is specific to mosquitoes. This study, combined with our previous analysis of dop1.462, suggests that RNAi insecticide technology provides a method for specifically targeting mosquito GPCRs without impacting GPCR activity in non-target species. Given that the proof of concept laboratory strain utilized in this study is not suitable for industry scale fermentations, future studies should aim to stably incorporate the 5-HTR.426 hairpin expression construct, which has been characterized here in a selectable plasmid-based laboratory yeast strain background, into a robust commercial-ready yeast strain that is suitable for scaled fermentation. The use of a commercial-ready strain would greatly decrease the cost of yeast production, as yeast would not need to be cultured in selective media, which is generally more expensive [44]. Inexpensive scaled yeast production would facilitate large-scale field trials in support of registry applications for this promising new pesticide class.

## Materials and methods

### Animal rearing

#### Indiana insectary

Mosquito strains used in this investigation were reared following established protocols^61^, with slight variations across different species. The strains used in the study include *A. aegypti* Liverpool-IB12 (LVP-IB12), *A. albopictus* Gainesville (BEI Resources, NIAID, NIH: MRA-804, donated by Sandra A. Allan), *A. gambiae* G3 (BEI Resources, NIAID, NIH: Eggs, MRA-112, provided by Mark Q. Benedict), *C. quinquefasciatus* JHB (supplied by the CDC to be distributed by BEI Resources, NIAID, NIH: Eggs, NR-43025) and *C. pipiens* which was established from ovitrap collection in Niles, MI, USA in 2021. These mosquitoes were reared within an insectary maintained at a temperature of 26.5 °C and an approximate relative humidity of 80%, and with either liver powder (900396, MP Biologicals, Solon, OH, USA; *A. aegypti* and *A. albopictus*), Koi fish food (71598 FS, Drs. Foster and Smith, Rhinelander, WI, USA; *A. gambiae*), or Hikari Cichlid pellets (Sinking Cichlid Gold, Hikari, Himeji, Japan; *C. quinquefasciatus* and *C. pipiens*). A 12-h light and dark cycle featuring 1-h crepuscular periods at the beginning and end of each cycle was implemented. The adults were provided 10% sucrose solution throughout their life cycles, and the adult females were blood fed via an artificial membrane feeding system (Hemotek Limited, Blackburn, UK) with defibrinated sheep blood (HemoStat Laboratories located in Dixon, CA, USA).

#### Trinidad insectary

*A. aegypti*, *A. albopictus* and *C. quinquefasciatus* collected in Trinidad (Diego Martin, Maraval and St. Augustine) were used to establish laboratory colonies. Larvae used were approximately fifth generation onward. All insectary activities were conducted at ambient temperature and humidity conditions in Trinidad. Adult female mosquitoes were blood fed as described above with fresh sheep’s blood (School of Veterinary Medicine, Faculty of Medical Sciences, Mt. Hope, UWI).

### Phylogenetic tree construction

A phylogenetic tree for *A. aegypti* AAEL000528 was generated using a method similar to Xu et al.^40^. The protein sequence of AAEL000528 was obtained from VectorBase^62^, and BLASTP was employed on blast.ncbi.nlm.nih.gov to identify sequences with similarity to the AAEL000528 protein. Accession numbers for receptors from each species are listed in Supplementary Table S2. The protein sequences were aligned using the CLUSTAL W algorithm within MEGA software version 11^63^. The “Find best DNA/Protein Model (ML)” function determined the appropriate model to compare the aligned sequences, leading to the selection of the Jones-Taylor-Thornton (JTT) model. The Maximum Likelihood method was employed with 1000-fold bootstrap replicates. The resulting bootstrap values are displayed above each node of the branches. The branch length, represented by a scale bar of 0.5 units, indicates the number of amino acid substitutions per sequence site. The *Ischnura elegans* 5-hydroxytryptamine receptor 1 (XP_046384840.1) was chosen to serve as an outgroup.

### Initial discovery of shRNA426

Detailed methodology for the screen in which siRNA #426 (a screen hit which has not yet been described), hereafter referred to as 5-HTR.426, was included in Hapairai et al.^48^. In the screen, soaking experiments with 5-HTR.426 siRNA, which were performed in both *A. aegypti* and *A. gambiae,* were conducted in replicate experiments, each involving 20 first instar larvae (L1). The larvae were exposed to 20 μL of 0.5 μg/μL 5-HTR.426 siRNA in a microfuge tube for a duration of four hours. Subsequently, the larvae were transferred to 50 mL sterile distilled water, reared under normal laboratory conditions, and evaluated following the larvicide testing guidelines established by the World Health Organization^64^, with the experiment concluding when the mosquitoes either die or reach adulthood. The larval screen data were analyzed using the Fisher’s exact test.

To assess the adulticidal potential of 5-HTR siRNA, a microinjection screen was performed using *A. aegypti* and *A. gambiae* as described by Hapairai et al.^19^. The microinjection experiments were conducted on adult female mosquitoes using an embryo microinjection protocol^65^ that was modified for adult female injections. Specifically, non-blood fed 3-day old females were anesthetized with carbon dioxide and injected with a needle prepared as described^65^ in the thoracic region with a dose of 2.25 μg of control or 5-HTR.426 siRNA (250 nL of 9 μg/μL for *A. aegypti* and 150 mL of 6 μg/μL for *A. gambiae* 5-HTR.426 siRNA) in a vertical orientation relative to the body axis. After the injection, the mosquitoes were placed in a cage for recovery and subsequently reared under normal laboratory conditions while being observed for behavioral abnormalities and mortality daily over the course of 1 week. In each of the three replicate experiments, 20 adult females were injected per treatment, and the data were analyzed using the Fisher’s exact test.

### RNAi yeast larvicide development and preparation

The 5-HTR.426 yeast strain was prepared as described by Mysore et al.^66^. In summary, custom-synthesized DNA oligonucleotides corresponding to the 5-HTR1 siRNA were obtained from Invitrogen Life Technologies (Carlsbad, CA, USA). The oligonucleotides were then cloned into the non-integrating pRS426 GPD yeast shuttle vector, which features a *URA3* marker and enables constitutive expression of the inserted sequences downstream of a *GPD *promoter, as previously detailed by Mumberg et al.^67^. The sequence of the hairpin expression cassette was verified by sequencing, and *S. cerevisiae* strain BY4742 (genotype *MATα his3Δ1 leu2Δ0 lys2Δ0 ura3Δ0*)^68^ was transformed with the plasmid, with transformants selected through growth on uracil-deficient minimal media. A control yeast strain prepared in this manner was previously described^48^ and used in this investigation. The expression of shRNA was confirmed in each strain through RT-PCR as previously outlined^41^. The yeasts were cultured and heat-killed as detailed by Mysore et al.^66^, and pelleted yeast were then lyophilized with a Labconco FreeZone 6 L Console Freeze Dryer (Labconco, Kansas City, MO, USA) as described by Mysore et al.^43^. The lyophilized yeast was utilized in larvicide and adulticide assays following the procedures described below.

### Larvicide experiments

#### Laboratory trials

5-HTR.426 yeast larvicide was evaluated following the WHO testing guidelines^69^ as outlined in Mysore et al.^66^. These evaluations were conducted on L1 laboratory strain larvae of all six species noted above. For each species, nine replicate container trials were conducted, with each trial utilizing 20 first-instar larvae (n = 180 larvae in total per treatment). The larvae were reared in 50 mL volumes of autoclaved distilled water placed in 500 mL containers. 40 mg of lyophilized yeast powder (either 5-HTR.426 or control) was added to each container at the beginning of each trial. The larvae were monitored throughout the trial period. At the end of the trial, the percentages of larval mortality were recorded and transformed using the arcsine transformation prior to analyzing the data using the Student’s t-test. Dose–response curves were generated and analyzed as previously described^48^ using different doses of larvicide prepared by using varying amounts of insecticidal and control interfering RNA yeast, which were then tested on the larvae. IBM SPSS Statistics (Version 29) was used to perform probit analyses.

*Semi-field larvicide trials:* Semi-field larvicide trials were conducted in a 2.44 m × 2.44 m tent enclosure with mesh sidewalls for aeration that were accessed through zipped openings between the months of March through July 2023. The enclosure was located on the roof of the Natural Sciences Building, Faculty of Science and Technology, University of the West Indies (UWI), Trinidad and Tobago (10°38′29.2″ N 61°24′02.2″ W). The maximum mean temperature during the experiment was 32.0 °C and the relative humidity ranged from 34 to 87% during those months.

Semi-field trial procedures were based on World Health Organization larvicide testing guidelines^64^. Briefly, 10 L experimental containers with 3.75 L of autoclaved reverse osmosis water were used. Each biological replicate experiment consisted of three replicates. In each container, 40 mg of larvicidal or control yeast was applied, and 20 newly hatched first-instar field strain larvae were added. Mortality was recorded each day until all surviving larvae reached adulthood. At the end of the trial, the percentages of larval mortality were transformed using the arcsine transformation prior to analyzing the data using the Student’s t-test.

### Yeast ATSB trials

#### Laboratory trial feeder system

The yeast Attractive Toxic Sugar Bait (ATSB) assays were performed as described in Mysore et al.^43^. Briefly, ATSB solution was prepared with 40 mg of lyophilized yeast (5-HTR.426 or control) containing 0.1% benzoic acid that was mixed with 100 μL of a 0.05% gellan gum and 5% sucrose stock solution. The feeders were prepared using a cut microfuge tube and placed in the test cages as described^43^. For *A. albopictus*, *C. quinquefasciatus*, and *C. pipiens*, 25 non-blood-fed adult females, aged 5–6 days, were selected. For *A. gambiae* and *A. aegypti*, 20 non-blood-fed adult females, aged 5–6 days, were chosen. These mosquitoes had been deprived of sugar for 24 h (*A. gambiae*) or 48 h (*A. aegypti*, *A. albopictus*, *C. quinquefasciatus*, and *C. pipiens*). The mosquitoes were allowed to feed for four hours from two feeders placed in 3.75 L insect cages. After the feeding period, engorgement was confirmed, and individuals that had not fed were removed from the cage. Daily survival rates were recorded for six days. These trials were performed in triplicate. The feeding rates were evaluated using the G-test of independence, and survival rates were compared using ANOVA in IBM SPSS Statistics (Version 29).

#### Miniature bait station sachet system used in simulated field studies in the laboratory and in semi-field trials conducted in Trinidad

Lyophilized yeast (5-HTR.426 or control) containing 40 mg and 0.1% benzoic acid, was combined with 100 μL of Westham Inc Bait (provided by Westham Co., Tel Aviv, Israel). This mixture was placed on a 1.3 cm square of thick plastic sheet and covered with a membrane supplied by Westham Co. The amount of yeast provided in each sachet was reduced to 4 mg for the outdoor semi-field *Aedes* mosquito trials. The membrane was sealed using a heat sealer to create a miniature membrane feeder (sachet). These sachets were placed either at the bottom of the cage for the simulated field studies in the lab or in a vertical position on the wall of the experimental cage (for outdoor semi-field trials) with the Westham Co. membrane facing inward to facilitate feeding. Simulated field trials using the sachets were conducted in the Indiana University insectary using laboratory strain mosquitoes, while all semi-field experiments in Trinidad were conducted on field strain mosquitoes in the tent enclosure described above during December 2022-January 2023 and June to September 2023. Three-day old adult females of each species were selected and sugar-starved for 24 h in the field setting described above and then allowed to feed for a period of 24 h. Water was provided for the duration of the experiment. Subsequently, the presence of engorged mosquitoes confirmed successful feeding. Daily survival rates were monitored over the next six days. Each experiment was conducted in triplicate. To assess the feeding rates, the G-test of independence was employed, while the survival rates were compared using ANOVA. Dose–response curves for adulticide 5-HTR.426 yeast ATSB were produced and evaluated as described in Mysore et al.^43^ with the lyophilized yeast and probit analyses performed using SPSS 29.

### Whole mount in situ hybridization

The Patel protocol^70^ was employed for the synthesis of a riboprobe corresponding to the *A. aegypti 5-HTR (AAEL000528)* gene. This riboprobe was subsequently utilized in in situ hybridization experiments performed on adult female mosquito brains following established procedures^71^. Three independent biological replicates were carried out using larvae that were fed with either control or 5-HTR.426 yeasts, as described previously. Following hybridizations, brain tissues were processed and mounted as described^72^. Examination of the brain tissues was conducted using a Zeiss Axio imager equipped with a Spot Flex camera (Diagnostic Instruments, Inc., Sterling Heights, MI, USA). For assessing the average signal intensity across the selected brain area, FIJI ImageJ software^73^ was employed to determine the mean gray values of digoxigenin-labeled transcript signals in the mosquitoes treated with 5-HTR.426 or control interfering RNA yeast. The resulting transcript data were analyzed using the Student's t-test.

### Determination of the mode of action

Immunohistochemical staining experiments were conducted on the brains of *A. aegypti* L4 larvae and adult mosquitoes subjected to treatments of either control or 5-HTR.426 yeast. The established methodologies outlined in previous studies^59,65^ were followed. The experiments involved the use of mAb nc82 anti-Bruchpilot antibody^58^ (DSHB Hybridoma Product nc82, deposited by E. Buchner to the DSHB), rabbit anti-horseradish peroxidase (HRP; 1:500, AbCam), and TO-PRO-3 iodide dye (Molecular Probes, Eugene, OR). Each experiment was performed in triplicate, utilizing brains from 20 L4 larvae or adults per treatment group. Following the completion of immunohistochemical processing, the brain tissues were mounted and subjected to high-resolution imaging using a Zeiss 710 confocal microscope equipped with Zen software. The acquired images were subsequently subjected to analysis employing FIJI ImageJ^73^ and Adobe Photoshop CC 2023 software. This facilitated the quantification of mean gray values, which represent the average signal intensities over the selected regions of interest. The calculation of mean gray values adhered to the prescribed methodology^72^. The data from both the control and 5-HTR.426 treatment groups were combined and underwent statistical analysis employing a two-tailed t-test with equal variance.

Serotonergic neurons were selectively labeled following the protocols outlined in Mysore et al.^59,74^. Briefly, rat anti-5HT antibody (1:100; Abcam, Cambridge, MA) was employed to mark serotonergic neurons in L4 larvae exposed to either 5-HTR.426 or control yeast. TO-PRO dye was used to label all cell bodies in the brain. The labeled brains were then imaged with a Zeiss 710 confocal microscope, and the resulting scanned images were analyzed using FIJI ImageJ^73^ and Adobe Photoshop CC 2023 software.

### Toxicity studies in non-target organisms

The toxicity assessment of 5-HTR.426 yeast was conducted following the methodologies outlined in Hapairai et al.^19^ and Mysore et al.^41^ for *D. melanogaster* and *T. castaneum,* respectively. To evaluate toxicity in *O. fasciatus* and *H. convergens* the following procedures were employed. Live adult *O. fasciatus* sourced from Carolina Biologicals (Burlington, NC, USA) were maintained under specified culture conditions. For the toxicity tests, conducted in duplicate, a slurry containing 200 μL of 10% sucrose mixed with red marker dye, along with 50 mg of either 5-HTR.426 or control interfering RNA yeast, was administered to 20 adults, ensuring that the total yeast consumption per insect matched that of the mosquito assays. To facilitate continuous delivery of the slurry over a six-day trial period, a 0.5 mL tube equipped with a wick was suspended from the cage, which was maintained at a room temperature of 21 °C. Verification of feeding was determined by observing feeding bouts and detecting red marker dye in the insect feces^42,43^. The survival data were analyzed using Fisher’s exact test. Similarly, *H. convergens* adults (obtained from Carolina Biologicals, Burlington, NC, USA) were reared in cages under room temperature conditions (21 °C) following supplier instructions. Toxicity assays were conducted as described above for *O. fasciatus*, with a cohort of 10 insects feeding on yeast ATSB provided in a small dish throughout the trial period.

### Supplementary Information

Supplementary Information.

## Data Availability

All data is available within the text and supplementary information supplied for this article.
