# Supplementary material for: Characterization of a novel RNAi yeast insecticide that silences mosquito 5-HT1 receptor genes
Source: Sci Rep. 2023 Dec 15;13:22511. doi: 10.1038/s41598-023-49799-3 (PMC10728091; doi:10.1038/s41598-023-49799-3)
Supplement: Supplementary file 1 — Supplementary Information. [file 41598_2023_49799_MOESM1_ESM.pdf]

Species/Taxon Group	Gene/Contig
Aedes aegypti	AAEL000528
Aedes albopictus	AALFPA_071742, AALFPA_054864, AALC636_034968
Anopheles arabiensis	AARA006243, AARA21_003352
Anopheles coluzzii	ACON011320, ACOM038010, ACMO_012652
Anopheles christyi	KB684830
Anopheles dirus	ADIR002152
Anopheles epiroticus	AEPI010697
Anopheles farauti	AFAF018411
Anopheles gambiae	AGAP011320
Anopheles melas	AMEC000482
Anopheles merus	AMEM006279, AMEM21_012446
Anopheles quadriannulatus	AQUA000037
Culex pipiens pallens	None
Culex quinquefasciatus	CPIJ017421, CQUJHB008800
Amphibians	None detected
Birds	None detected
Fish	None detected
Fungi	None detected
Human	None detected
Mammals	None detected
Plants	None detected
Reptiles	None detected

Supplementary Table S1. 5HT-R.426 target site conservation. *Aedes, Anopheles,* and *Culex* mosquito genes containing the 5HT-R.426 target site (100% identity) are shown. The target site has not yet been identified outside of mosquitoes and was not detected in the other indicated taxa.

Sl. No	Description	Scientific Name	Common Name	Query Cover	E value	Per. ident	Acc. Len	Accession
1	5-hydroxytryptamine receptor 1D	*Aedes aegypti*	yellow fever mosquito	100%	0	100	566	XP_001647960.3
2	5-hydroxytryptamine receptor 1D	*Aedes albopictus*	Asian tiger mosquito	100%	0	92.95	561	XP_019931072.2
3	5-hydroxytryptamine receptor 1D-like	*Aedes albopictus*	Asian tiger mosquito	100%	0	91.89	561	XP_029731706.1
4	5-hydroxytryptamine receptor 1D	*Sabethes cyaneus*	NA	99%	0	77.6	565	XP_053688407.1
5	5-hydroxytryptamine receptor 1D isoform X1	*Wyeomyia smithii*	pitcher-plant mosquito	99%	0	78.65	564	XP_055536337.1
6	trace amine-associated receptor 1	*Culex quinquefasciatus*	southern house mosquito	99%	0	77.62	554	XP_038112473.1
7	5-hydroxytryptamine receptor 1A	*Toxorhynchites rutilus septentrionalis*	NA	99%	0	80.74	555	XP_055627983.1
8	trace amine-associated receptor 1	*Culex pipiens pallens*	northern house mosquito	99%	0	77.45	554	XP_039446393.1
9	5-hydroxytryptamine receptor 1 isoform X2	*Wyeomyia smithii*	pitcher-plant mosquito	99%	0	74.65	540	XP_055536339.1
10	5-hydroxytryptamine receptor 1A	*Anopheles maculipalpis*	NA	99%	0	71.01	580	XP_050067147.1
11	5-hydroxytryptamine receptor 1A	*Anopheles marshallii*	NA	99%	0	70.59	586	XP_053669126.1
12	5-hydroxytryptamine receptor 1D	*Anopheles darlingi*	NA	99%	0	68.75	601	XP_049542467.1
13	5-hydroxytryptamine receptor 1A	*Anopheles moucheti*	mosquito	99%	0	69.85	586	XP_052889247.1
14	5-hydroxytryptamine receptor 1A	*Anopheles stephensi*	Asian malaria mosquito	99%	0	71.28	584	XP_035894269.1
15	5-hydroxytryptamine receptor 1	*Anopheles arabiensis*	NA	99%	0	69.69	575	XP_040167376.1
16	5-hydroxytryptamine receptor 1	*Anopheles coluzzii*	NA	99%	0	69.86	575	XP_040237356.2
17	5-hydroxytryptamine receptor 1	*Anopheles merus*	NA	99%	0	69.69	575	XP_041781405.1
18	5-hydroxytryptamine receptor 1A	*Anopheles funestus*	African malaria mosquito	99%	0	69.54	583	XP_049298398.1
19	5-hydroxytryptamine receptor 1D	*Anopheles cruzii*	mosquito	99%	0	69.83	576	XP_052860781.1
20	5-hydroxytryptamine receptor 1A	*Anopheles nili*	NA	99%	0	71.87	573	XP_053671050.1
21	5-hydroxytryptamine receptor 1A	*Anopheles funestus*	African malaria mosquito	99%	0	69.43	584	XP_049298396.1
22	5-hydroxytryptamine receptor 1D-like	*Anopheles albimanus*	NA	99%	0	69.85	594	XP_035785223.1
23	5-hydroxytryptamine receptor 1A	*Anopheles aquasalis*	NA	99%	0	68.8	608	XP_050090062.1
24	AGAP011320-PA	*Anopheles gambiae str. PEST*	NA	75%	0	69.81	467	XP_564170.3
25	5-hydroxytryptamine receptor 1D	*Sitodiplosis mosellana*	orange wheat blossom midge	99%	1.00E-169	47.23	564	XP_055312764.1
26	5-hydroxytryptamine receptor 1D isoform X2	*Glossina fuscipes*	NA	97%	4.00E-166	45.92	591	XP_037883365.1
27	5-hydroxytryptamine receptor 1D isoform X1	*Glossina fuscipes*	NA	97%	8.00E-166	45.92	608	XP_037883364.1
28	trace amine-associated receptor 1	*Contarinia nasturtii*	swede midge	98%	1.00E-165	47.01	563	XP_031616846.1
29	trace amine-associated receptor 1 isoform X2	*Lutzomyia longipalpis*	NA	97%	1.00E-165	48.92	458	XP_055691349.1
30	trace amine-associated receptor 1 isoform X1	*Lutzomyia longipalpis*	NA	97%	3.00E-165	48.83	470	XP_055691346.1
31	trace amine-associated receptor 1	*Phlebotomus papatasi*	NA	99%	7.00E-164	48.43	464	XP_055705323.1
32	uncharacterized protein LOC108978771	*Bactrocera latifrons*	NA	93%	5.00E-163	58.41	737	XP_018804761.1
33	beta-3 adrenergic receptor isoform X1	*Episyrphus balteatus*	marmalade hoverfly	97%	2.00E-162	48.72	682	XP_055856352.1
34	trace amine-associated receptor 1 isoform X3	*Glossina fuscipes*	NA	84%	3.00E-162	48.98	544	XP_037883368.1
35	beta-3 adrenergic receptor isoform X3	*Condylostylus longicornis*	NA	96%	7.00E-161	45.95	610	XP_055390339.1
36	beta-3 adrenergic receptor isoform X2	*Condylostylus longicornis*	NA	84%	1.00E-160	52.49	669	XP_055390338.1
37	beta-3 adrenergic receptor isoform X1	*Condylostylus longicornis*	NA	84%	2.00E-160	52.49	681	XP_055390337.1
38	beta-3 adrenergic receptor	*Scaptodrosophila lebanonensis*	NA	72%	5.00E-157	58.61	648	XP_030379210.1
39	5-hydroxytryptamine receptor 1D	*Drosophila innubila*	NA	74%	1.00E-156	57.7	649	XP_034478231.1
40	uncharacterized protein LOC6639693	*Drosophila willistoni*	NA	96%	7.00E-156	57.76	763	XP_023037660.1
41	uncharacterized protein Dwil_GK19464	*Drosophila willistoni*	NA	96%	9.00E-156	57.76	745	EDW73861.2
42	trace amine-associated receptor 1	*Eupeodes corollae*	NA	97%	9.00E-156	46.6	561	XP_055924046.1
43	5-hydroxytryptamine receptor 1A	*Drosophila navojoa*	NA	74%	1.00E-155	56.76	670	XP_030243274.1

44	5-hydroxytryptamine receptor 1A	*Drosophila virilis*	NA	72%	7.00E-155	59.15	657	XP_002049478.3
45	uncharacterized protein Dvir_GJ21608	*Drosophila virilis*	NA	72%	2.00E-154	59.15	731	EDW60671.2
46	5-hydroxytryptamine receptor 1B	*Drosophila novamexicana*	NA	72%	5.00E-154	58.41	733	XP_030554184.1
47	5-hydroxytryptamine receptor 1B	*Ceratitis capitata*	Mediterranean fruit fly	93%	1.00E-153	55.86	707	XP_023158891.1
48	hypothetical protein AWZ03_010529	*Drosophila navojoa*	NA	74%	4.00E-148	55.41	662	TDG43065.1
49	trace amine-associated receptor 1	*Anoplophora glabripennis*	Asian longhorned beetle	82%	2.00E-145	48.93	443	XP_023309704.1
50	trace amine-associated receptor 1 isoform X1	*Diorhabda sublineata*	NA	81%	4.00E-143	47.65	437	XP_056635471.1
51	hypothetical protein HA402_004697	*Bradysia odoriphaga*	NA	99%	5.00E-139	45.53	480	KAG4072608.1
52	trace amine-associated receptor 1 isoform X2	*Bradysia coprophila*	NA	99%	1.00E-138	44.76	476	XP_037052483.1
53	trace amine-associated receptor 1 isoform X1	*Bradysia coprophila*	NA	99%	2.00E-138	44.76	480	XP_037052482.1
54	5-hydroxytryptamine receptor 1A isoform X2	*Frankliniella occidentalis*	western flower thrips	65%	2.00E-137	53.27	667	XP_052124421.1
55	5-hydroxytryptamine receptor 1A isoform X1	*Frankliniella occidentalis*	western flower thrips	70%	3.00E-137	51.13	668	XP_052124420.1
56	trace amine-associated receptor 9 isoform X2	*Neodiprion lecontei*	redheaded pine sawfly	74%	3.00E-134	49.67	417	XP_046599215.1
57	trace amine-associated receptor 9 isoform X2	*Neodiprion pinetum*	white pine sawfly	74%	4.00E-134	49.67	421	XP_046486428.1
58	trace amine-associated receptor 1	*Tribolium madens*	black flour beetle	82%	3.00E-133	47.87	422	XP_044258936.1
59	5-hydroxytryptamine receptor 1	*Ischnura elegans*	NA	77%	4.00E-131	45.35	668	XP_046384840.1
60	trace amine-associated receptor 1 isoform X2	*Megachile rotundata*	alfalfa leafcutting bee	68%	7.00E-128	52.56	448	XP_012145344.1
61	trace amine-associated receptor 9	*Leptopilina boulardi*	NA	70%	7.00E-127	51.83	415	XP_051157115.1
62	hypothetical protein M5D96_006954	*Drosophila gunungcola*	fruit fly	92%	3.00E-120	50.48	678	KAI8039540.1
63	unnamed protein product	*Tenebrio molitor*	yellow mealworm	82%	4.00E-118	44.68	423	CAH1374049.1
64	trace amine-associated receptor 1 isoform X2	*Drosophila sechellia*	NA	41%	1.00E-116	74.36	344	XP_032571860.1
65	trace amine-associated receptor 1 isoform X2	*Drosophila mauritiana*	NA	41%	2.00E-116	74.36	350	XP_033155073.1
66	trace amine-associated receptor 1	*Aethina tumida*	small hive beetle	82%	2.00E-116	44.49	426	XP_019867795.1
67	5-hydroxytryptamine receptor 1D	*Drosophila erecta*	NA	41%	5.00E-114	74.36	650	XP_026836667.1
68	uncharacterized protein Dmel_CG13579, isoform A	*Drosophila melanogaster*	fruit fly	41%	5.00E-114	74.36	650	NP_611917.2
69	5-hydroxytryptamine receptor 1A	*Drosophila eugracilis*	NA	41%	5.00E-114	74.36	656	XP_017070508.2
70	5-hydroxytryptamine receptor 1D	*Drosophila yakuba*	NA	41%	6.00E-114	74.36	651	XP_002092825.3
71	5-hydroxytryptamine receptor 1A	*Drosophila hydei*	NA	42%	7.00E-114	72.73	665	XP_023163332.2
72	5-hydroxytryptamine receptor 1A isoform X1	*Drosophila sechellia*	NA	41%	7.00E-114	74.36	656	XP_032571859.1
73	5-hydroxytryptamine receptor 1D	*Drosophila simulans*	NA	41%	8.00E-114	74.36	654	XP_039147397.1
74	beta-3 adrenergic receptor	*Drosophila pseudoobscura*	NA	41%	8.00E-114	74.36	713	XP_033233813.1
75	5-hydroxytryptamine receptor 1D	*Drosophila santomea*	NA	41%	9.00E-114	74.36	654	XP_039482203.1
76	5-hydroxytryptamine receptor 1A isoform X1	*Drosophila mauritiana*	NA	41%	1.00E-113	74.36	662	XP_033155071.1
77	5-hydroxytryptamine receptor 1A	*Drosophila obscura*	NA	61%	1.00E-113	74.36	665	XP_041448068.1
78	5-hydroxytryptamine receptor 1A	*Drosophila mojavensis*	NA	42%	1.00E-113	72.73	681	XP_032585733.1
79	5-hydroxytryptamine receptor 1A	*Drosophila biarmipes*	NA	41%	1.00E-113	74.36	663	XP_016947805.2
80	5-hydroxytryptamine receptor 1A	*Drosophila arizonae*	NA	42%	1.00E-113	72.73	684	XP_017868595.1
81	5-hydroxytryptamine receptor 1A	*Drosophila persimilis*	NA	41%	1.00E-113	74.36	665	XP_026842348.1
82	hypothetical protein KR084_008731	*Drosophila pseudotakahashii*	NA	41%	1.00E-113	74.36	690	KAH8346380.1
83	5-hydroxytryptamine receptor 1A	*Drosophila miranda*	NA	41%	1.00E-113	74.36	666	XP_033247561.1
84	uncharacterized protein Dmel_CG13579, isoform B	*Drosophila melanogaster*	fruit fly	41%	2.00E-113	74.36	716	NP_001261169.1
85	5-hydroxytryptamine receptor 1A	*Drosophila guanche*	NA	41%	2.00E-113	74.36	672	XP_034133675.1
86	5-hydroxytryptamine receptor 1A isoform X1	*Drosophila albomicans*	NA	41%	2.00E-113	73.93	662	XP_051859939.1
87	5-hydroxytryptamine receptor 1A	*Drosophila grimshawi*	NA	41%	2.00E-113	73.93	676	XP_032591144.2

88	beta-3 adrenergic receptor	*Drosophila ananassae*	NA	41%	2.00E-113	74.36	662	XP_001960942.3
89	uncharacterized protein Dere_GG22970	*Drosophila erecta*	NA	41%	2.00E-113	74.36	716	EDV57004.2
90	alpha-protein kinase 1	*Drosophila subobscura*	NA	41%	2.00E-113	74.36	674	XP_034652461.1
91	5-hydroxytryptamine receptor 1A	*Drosophila ficusphila*	NA	41%	2.00E-113	74.36	664	XP_017062004.1
92	uncharacterized protein Dsimw501_GD11861	*Drosophila simulans*	NA	41%	3.00E-113	74.36	720	KMY96336.1
93	uncharacterized protein LOC115066067	*Bactrocera dorsalis*	oriental fruit fly	84%	3.00E-113	65.34	762	XP_049309876.1
94	5-hydroxytryptamine receptor 1A	*Drosophila takahashii*	NA	41%	3.00E-113	74.36	735	XP_017000716.2
95	uncharacterized protein LOC126756675	*Bactrocera neohumeralis*	NA	65%	3.00E-113	65.34	760	XP_050325869.1
96	GH21002	*Drosophila grimshawi*	NA	41%	4.00E-113	73.93	706	EDW00658.1
97	uncharacterized protein Dmoj_GI20641	*Drosophila mojavensis*	NA	42%	4.00E-113	72.73	755	EDW09690.2
98	GM11863	*Drosophila sechellia*	NA	41%	4.00E-113	74.36	712	EDW49194.1
99	GL20140	*Drosophila persimilis*	NA	41%	5.00E-113	73.93	657	EDW33293.1
100	hypothetical protein KR067_007703	*Drosophila pandora*	NA	41%	5.00E-113	74.36	696	KAH8340067.1

Supplementary Table S2. 5HT-R.426 BLASTP comparison results. The *A. aegypti* AAEL000528 protein sequence (XP_001647960.3-highlighted in yellow) retrieved from VectorBase was used to obtain the above table of similar protein sequences from various invertebrate species. These sequences were used to perform the phylogenetic studies outlined in the methods section, the results of which are shown in Supplementary Fig. S2. The sequence from *Ischnura elegans* (highlighted in green) was used as the outlier in these analyses.

NCBI Multiple Sequence Alignment Viewer, Version 1.25.0

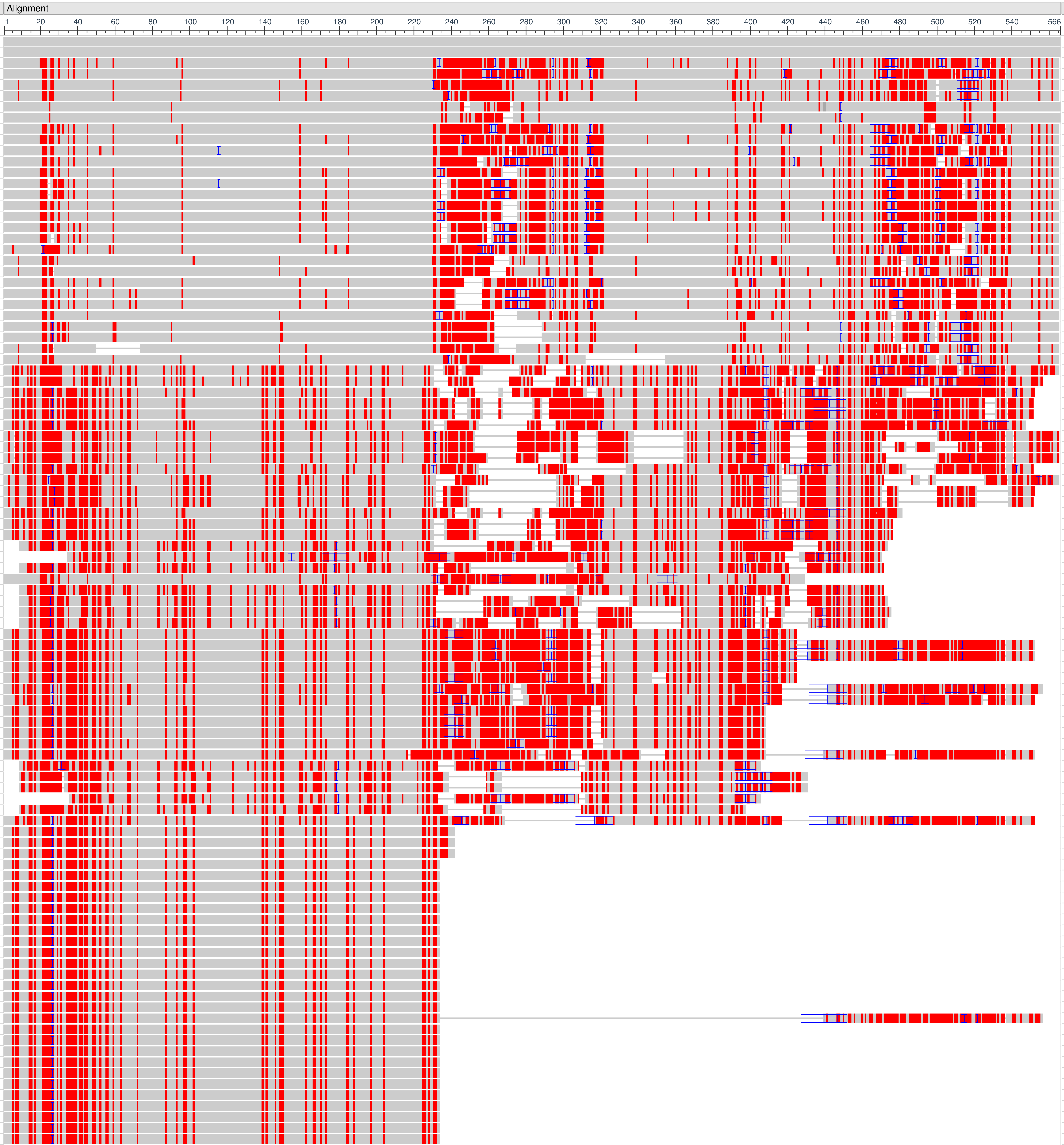

Sequence ID	Start	End	Organism
Query_31991 (+)	1	566	
XP_001647960.3 (+)	1	566	Aedes aegypti
XP_035894269.1 (+)	1	583	Anopheles stephensi
XP_050090062.1 (+)	1	607	Anopheles aquasalis
XP_058830879.1 (+)	1	579	Topomyia yanbarensis
XP_058463405.1 (+)	1	578	Malaya genurostris
XP_019931072.2 (+)	1	561	Aedes albopictus
XP_029731706.1 (+)	1	561	Aedes albopictus
XP_035785223.1 (+)	1	593	Anopheles albimanus
XP_050067147.1 (+)	1	579	Anopheles maculipalpis
XP_058067019.1 (+)	1	587	Anopheles bellator
XP_049542467.1 (+)	1	600	Anopheles darlingi
XP_040237356.2 (+)	1	574	Anopheles coluzzii
XP_049298396.1 (+)	1	583	Anopheles funestus
XP_049298398.1 (+)	1	582	Anopheles funestus
XP_041781405.1 (+)	1	574	Anopheles merus
XP_040167376.1 (+)	1	574	Anopheles arabiensis
XP_053669126.1 (+)	1	585	Anopheles marshallii
XP_052889247.1 (+)	1	585	Anopheles moucheti
XP_053671050.1 (+)	1	572	Anopheles nili
XP_053688407.1 (+)	1	564	Sabethes cyaneus
XP_055536337.1 (+)	1	563	Wyeomyia smithii
XP_052860781.1 (+)	1	575	Anopheles cruzii
XP_058117881.1 (+)	1	575	Anopheles coustani
XP_058172676.1 (+)	1	577	Anopheles ziemanni
XP_055627983.1 (+)	3	554	Toxorhynchites rutilus se...
XP_039446393.1 (+)	1	553	Culex pipiens pallens
XP_038112473.1 (+)	1	553	Culex quinquefasciatus
XP_055536339.1 (+)	1	539	Wyeomyia smithii
XP_058463406.1 (+)	1	535	Malaya genurostris
XP_055312764.1 (+)	1	563	Sitodiplosis mosellana
XP_031616846.1 (+)	1	554	Contarinia nasturtii
XP_055856352.1 (+)	111	650	Episyrphus balteatus
XP_037883365.1 (+)	1	565	Glossina fuscipes
XP_037883364.1 (+)	18	582	Glossina fuscipes
XP_055390339.1 (+)	13	575	Condylostylus longicornis
XP_037052482.1 (+)	1	472	Bradysia coprophila
KAG4072608.1 (+)	1	472	Bradysia odoriphaga
XP_037052483.1 (+)	1	468	Bradysia coprophila
XP_055924046.1 (+)	1	506	Eupeodes corollae
XP_055705323.1 (+)	1	463	Phlebotomus papatasi
XP_055691346.1 (+)	1	452	Lutzomyia longipalpis
XP_055691349.1 (+)	1	451	Lutzomyia longipalpis
XP_037883368.1 (+)	18	517	Glossina fuscipes
XP_055390338.1 (+)	1	463	Condylostylus longicornis
XP_055390337.1 (+)	13	475	Condylostylus longicornis
XP_023309704.1 (+)	8	420	Anoplophora glabripennis
XP_046384840.1 (+)	61	579	Ischnura elegans
XP_057652217.1 (+)	12	412	Diorhabda carinulata
XP_564170.3 (+)	1	467	Anopheles gambiae str. ...
XP_056635471.1 (+)	12	412	Diorhabda sublineata
XP_044258936.1 (+)	8	399	Tribolium madens
CAH1374049.1 (+)	8	402	Tenebrio molitor
XP_019867795.1 (+)	8	403	Aethina tumida
XP_030243274.1 (+)	1	437	Drosophila navojoa
EDW73861.2 (+)	1	636	Drosophila willistoni
XP_023037660.1 (+)	19	654	Drosophila willistoni
XP_034478231.1 (+)	1	428	Drosophila innubila
TDG43065.1 (+)	1	429	Drosophila navojoa
XP_023158891.1 (+)	6	677	Ceratitis capitata
XP_018804761.1 (+)	1	646	Bactrocera latifrons
XP_030554184.1 (+)	1	422	Drosophila novamexicana
EDW60671.2 (+)	1	420	Drosophila virilis
XP_002049478.3 (+)	1	420	Drosophila virilis
XP_030379210.1 (+)	1	412	Scaptodrosophila lebano...
KAI8039540.1 (+)	1	598	Drosophila gunungcola
XP_052124420.1 (+)	13	444	Frankliniella occidentalis
XP_046599215.1 (+)	9	393	Neodiprion lecontei
XP_046486428.1 (+)	9	393	Neodiprion pinetum
XP_052124421.1 (+)	43	443	Frankliniella occidentalis
XP_012145344.1 (+)	47	373	Megachile rotundata
XP_049309876.1 (+)	1	723	Bactrocera dorsalis
XP_032585733.1 (+)	1	242	Drosophila mojavensis
XP_017868595.1 (+)	1	242	Drosophila arizonae
XP_023163332.2 (+)	1	242	Drosophila hydei
XP_034652461.1 (+)	1	234	Drosophila subobscura
EDV57004.2 (+)	1	234	Drosophila erecta
XP_001960942.3 (+)	1	234	Drosophila ananassae
XP_032591144.2 (+)	9	242	Drosophila grimshawi
XP_051859939.1 (+)	1	234	Drosophila albomicans
XP_034133675.1 (+)	1	234	Drosophila guanche
NP_001261169.1 (+)	1	234	Drosophila melanogaster
XP_017062004.1 (+)	1	234	Drosophila ficusphila
XP_033247561.1 (+)	1	234	Drosophila miranda
KAH8346380.1 (+)	1	234	Drosophila pseudotakah...
XP_026842348.1 (+)	1	234	Drosophila persimilis
KMY96336.1 (+)	1	234	Drosophila simulans
XP_016947805.2 (+)	1	234	Drosophila biarmipes
XP_017000716.2 (+)	1	234	Drosophila takahashii
XP_041448068.1 (+)	1	646	Drosophila obscura
XP_032571860.1 (+)	1	234	Drosophila sechellia
XP_033155073.1 (+)	1	234	Drosophila mauritiana
XP_026836667.1 (+)	1	234	Drosophila erecta
NP_611917.2 (+)	1	234	Drosophila melanogaster
XP_017070508.2 (+)	1	234	Drosophila eugracilis
XP_002092825.3 (+)	1	234	Drosophila yakuba
XP_032571859.1 (+)	1	234	Drosophila sechellia
XP_039147397.1 (+)	1	234	Drosophila simulans
XP_033233813.1 (+)	53	286	Drosophila pseudoobscura
XP_039482203.1 (+)	1	234	Drosophila santomea
XP_033155071.1 (+)	1	234	Drosophila mauritiana

Supplementary Fig. S1. 5-HTR1 protein sequence alignment. Protein sequences aligned in NCBI blastp were viewed using the NCBI Multiple Sequence Alignment Viewer, Version 1.25.0. Mismatches are highlighted in red and gray indicates consensus sequences when compared with the AAEL000528 protein query sequence (highlighted in yellow). The *I. elegans* 5-HTR1 protein, which was used as an outgroup in subsequent phylogenetic analyses, is highlighted in green.

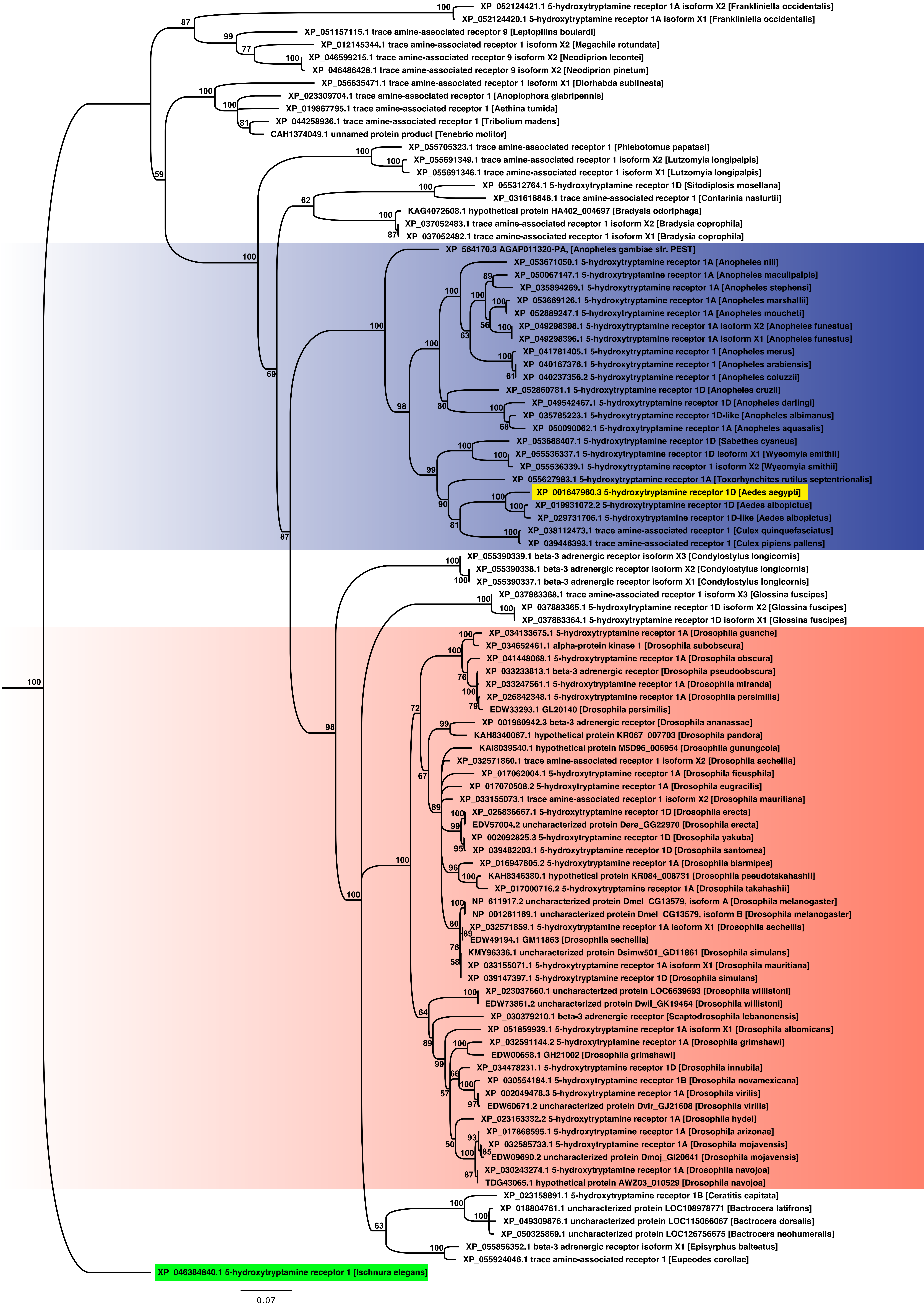

XP_052124421.1 5-hydroxytryptamine receptor 1A isoform X2 [Frankliniella occidentalis]
XP_052124420.1 5-hydroxytryptamine receptor 1A isoform X1 [Frankliniella occidentalis]
XP_051157115.1 trace amine-associated receptor 9 [Leptopilina boulardi]
XP_012145344.1 trace amine-associated receptor 1 isoform X2 [Megachile rotundata]
XP_046599215.1 trace amine-associated receptor 9 isoform X2 [Neodiprion lecontei]
XP_046486428.1 trace amine-associated receptor 9 isoform X2 [Neodiprion pinetum]
XP_056635471.1 trace amine-associated receptor 1 isoform X1 [Diorhabda sublineata]
XP_023309704.1 trace amine-associated receptor 1 [Anoplophora glabripennis]
XP_019867795.1 trace amine-associated receptor 1 [Aethina tumida]
XP_044258936.1 trace amine-associated receptor 1 [Tribolium madens]
CAH1374049.1 unnamed protein product [Tenebrio molitor]
XP_055705323.1 trace amine-associated receptor 1 [Phlebotomus papatasi]
XP_055691349.1 trace amine-associated receptor 1 isoform X2 [Lutzomyia longipalpis]
XP_055691346.1 trace amine-associated receptor 1 isoform X1 [Lutzomyia longipalpis]
XP_055312764.1 5-hydroxytryptamine receptor 1D [Sitodiplosis mosellana]
XP_031616846.1 trace amine-associated receptor 1 [Contarinia nasturtii]
KAG4072608.1 hypothetical protein HA402_004697 [Bradysia odoriphaga]
XP_037052483.1 trace amine-associated receptor 1 isoform X2 [Bradysia coprophila]
XP_037052482.1 trace amine-associated receptor 1 isoform X1 [Bradysia coprophila]
XP_564170.3 AGAP011320-PA, [Anopheles gambiae str. PEST]
XP_053671050.1 5-hydroxytryptamine receptor 1A [Anopheles nili]
XP_050067147.1 5-hydroxytryptamine receptor 1A [Anopheles maculipalpis]
XP_035894269.1 5-hydroxytryptamine receptor 1A [Anopheles stephensi]
XP_053669126.1 5-hydroxytryptamine receptor 1A [Anopheles marshallii]
XP_052889247.1 5-hydroxytryptamine receptor 1A [Anopheles moucheti]
XP_049298398.1 5-hydroxytryptamine receptor 1A isoform X2 [Anopheles funestus]
XP_049298396.1 5-hydroxytryptamine receptor 1A isoform X1 [Anopheles funestus]
XP_041781405.1 5-hydroxytryptamine receptor 1 [Anopheles merus]
XP_040167376.1 5-hydroxytryptamine receptor 1 [Anopheles arabiensis]
XP_040237356.2 5-hydroxytryptamine receptor 1 [Anopheles coluzzii]
XP_052860781.1 5-hydroxytryptamine receptor 1D [Anopheles cruzii]
XP_049542467.1 5-hydroxytryptamine receptor 1D [Anopheles darlingi]
XP_035785223.1 5-hydroxytryptamine receptor 1D-like [Anopheles albimanus]
XP_050090062.1 5-hydroxytryptamine receptor 1A [Anopheles aquasalis]
XP_053688407.1 5-hydroxytryptamine receptor 1D [Sabethes cyaneus]
XP_055536337.1 5-hydroxytryptamine receptor 1D isoform X1 [Wyeomyia smithii]
XP_055536339.1 5-hydroxytryptamine receptor 1 isoform X2 [Wyeomyia smithii]
XP_055627983.1 5-hydroxytryptamine receptor 1A [Toxorhynchites rutilus septentrionalis]
XP_001647960.3 5-hydroxytryptamine receptor 1D [Aedes aegypti]
XP_019931072.2 5-hydroxytryptamine receptor 1D [Aedes albopictus]
XP_029731706.1 5-hydroxytryptamine receptor 1D-like [Aedes albopictus]
XP_038112473.1 trace amine-associated receptor 1 [Culex quinquefasciatus]
XP_039446393.1 trace amine-associated receptor 1 [Culex pipiens pallens]
XP_055390339.1 beta-3 adrenergic receptor isoform X3 [Condylostylus longicornis]
XP_055390338.1 beta-3 adrenergic receptor isoform X2 [Condylostylus longicornis]
XP_055390337.1 beta-3 adrenergic receptor isoform X1 [Condylostylus longicornis]
XP_037883368.1 trace amine-associated receptor 1 isoform X3 [Glossina fuscipes]
XP_037883365.1 5-hydroxytryptamine receptor 1D isoform X2 [Glossina fuscipes]
XP_037883364.1 5-hydroxytryptamine receptor 1D isoform X1 [Glossina fuscipes]
XP_034133675.1 5-hydroxytryptamine receptor 1A [Drosophila guanche]
XP_034652461.1 alpha-protein kinase 1 [Drosophila subobscura]
XP_041448068.1 5-hydroxytryptamine receptor 1A [Drosophila obscura]
XP_033233813.1 beta-3 adrenergic receptor [Drosophila pseudoobscura]
XP_033247561.1 5-hydroxytryptamine receptor 1A [Drosophila miranda]
XP_026842348.1 5-hydroxytryptamine receptor 1A [Drosophila persimilis]
EDW33293.1 GL20140 [Drosophila persimilis]
XP_001960942.3 beta-3 adrenergic receptor [Drosophila ananassae]
KAH8340067.1 hypothetical protein KR067_007703 [Drosophila pandora]
KAI8039540.1 hypothetical protein M5D96_006954 [Drosophila gunungcola]
XP_032571860.1 trace amine-associated receptor 1 isoform X2 [Drosophila sechellia]
XP_017062004.1 5-hydroxytryptamine receptor 1A [Drosophila ficusphila]
XP_017070508.2 5-hydroxytryptamine receptor 1A [Drosophila eugracilis]
XP_033155073.1 trace amine-associated receptor 1 isoform X2 [Drosophila mauritiana]
XP_026836667.1 5-hydroxytryptamine receptor 1D [Drosophila erecta]
EDV57004.2 uncharacterized protein Dere_GG22970 [Drosophila erecta]
XP_002092825.3 5-hydroxytryptamine receptor 1D [Drosophila yakuba]
XP_039482203.1 5-hydroxytryptamine receptor 1D [Drosophila santomea]
XP_016947805.2 5-hydroxytryptamine receptor 1A [Drosophila biarmipes]
KAH8346380.1 hypothetical protein KR084_008731 [Drosophila pseudotakahashii]
XP_017000716.2 5-hydroxytryptamine receptor 1A [Drosophila takahashii]
NP_611917.2 uncharacterized protein Dmel_CG13579, isoform A [Drosophila melanogaster]
NP_001261169.1 uncharacterized protein Dmel_CG13579, isoform B [Drosophila melanogaster]
XP_032571859.1 5-hydroxytryptamine receptor 1A isoform X1 [Drosophila sechellia]
EDW49194.1 GM11863 [Drosophila sechellia]
KMY96336.1 uncharacterized protein Dsimw501_GD11861 [Drosophila simulans]
XP_033155071.1 5-hydroxytryptamine receptor 1A isoform X1 [Drosophila mauritiana]
XP_039147397.1 5-hydroxytryptamine receptor 1D [Drosophila simulans]
XP_023037660.1 uncharacterized protein LOC6639693 [Drosophila willistoni]
EDW73861.2 uncharacterized protein Dwil_GK19464 [Drosophila willistoni]
XP_030379210.1 beta-3 adrenergic receptor [Scaptodrosophila lebanonensis]
XP_051859939.1 5-hydroxytryptamine receptor 1A isoform X1 [Drosophila albomicans]
XP_032591144.2 5-hydroxytryptamine receptor 1A [Drosophila grimshawi]
EDW00658.1 GH21002 [Drosophila grimshawi]
XP_034478231.1 5-hydroxytryptamine receptor 1D [Drosophila innubila]
XP_030554184.1 5-hydroxytryptamine receptor 1B [Drosophila novamexicana]
XP_002049478.3 5-hydroxytryptamine receptor 1A [Drosophila virilis]
EDW60671.2 uncharacterized protein Dvir_GJ21608 [Drosophila virilis]
XP_023163332.2 5-hydroxytryptamine receptor 1A [Drosophila hydei]
XP_017868595.1 5-hydroxytryptamine receptor 1A [Drosophila arizonae]
XP_032585733.1 5-hydroxytryptamine receptor 1A [Drosophila mojavensis]
EDW09690.2 uncharacterized protein Dmoj_GI20641 [Drosophila mojavensis]
XP_030243274.1 5-hydroxytryptamine receptor 1A [Drosophila navojoa]
TDG43065.1 hypothetical protein AWZ03_010529 [Drosophila navojoa]
XP_023158891.1 5-hydroxytryptamine receptor 1B [Ceratitis capitata]
XP_018804761.1 uncharacterized protein LOC108978771 [Bactrocera latifrons]
XP_049309876.1 uncharacterized protein LOC115066067 [Bactrocera dorsalis]
XP_050325869.1 uncharacterized protein LOC126756675 [Bactrocera neohumeralis]
XP_055856352.1 beta-3 adrenergic receptor isoform X1 [Episyrphus balteatus]
XP_055924046.1 trace amine-associated receptor 1 [Eupeodes corollae]
XP_046384840.1 5-hydroxytryptamine receptor 1 [Ischnura elegans]
0.07

Supplementary Fig. S2: Phylogenetic tree of *A. aegypti* AAEL000528 protein. Phylogenetic tree, branch length and bootstrap values of serotonin receptors of various species obtained from BLASTN analyses are indicated. The numbers next to the branches represent the bootstrap values for each branch (1000 replications, significant support for the related sequences common to a node). Branch lengths are scaled. (scale bar indicates 0.07 units of branch length). European Bluetail (*I. elegans*) is used as the out group (green). The protein encoded by AAEL000528 is highlighted in yellow.
